# Design, Synthesis, and Biological Evaluation of [1,2,4]triazolo[4,3-*a*] Pyrazine Derivatives as Novel Dual c-Met/VEGFR-2 Inhibitors

**DOI:** 10.3389/fchem.2022.815534

**Published:** 2022-04-06

**Authors:** Xiaobo Liu, Yuzhen Li, Qian Zhang, Qingshan Pan, Pengwu Zheng, Xinyang Dai, Zhaoshi Bai, Wufu Zhu

**Affiliations:** ^1^ Jiangxi Provincial Key Laboratory of Drug Design and Evaluation, School of Pharmacy, Jiangxi Science and Technology Normal University, Nanchang, China; ^2^ School of Chemical Engineering, Dalian University of Technology, Dalian, China; ^3^ Jiangsu Cancer Hospital and Jiangsu Institute of Cancer Research and the Affiliated Cancer Hospital of Nanjing Medical University, Nanjing, China

**Keywords:** c-Met inhibitor, antiproliferative activity, antitumor, targeted drug, pyrazine derivatives

## Abstract

In this study, we designed and synthesized a series of novel [1,2,4]triazolo [4,3-*a*]pyrazine derivatives, and evaluated them for their inhibitory activities toward c-Met/VEGFR-2 kinases and antiproliferative activities against tested three cell lines *in vitro*. Most of the compounds showed satisfactory activity compared with lead compound foretinib. Among them, the most promising compound **17l** exhibited excellent antiproliferative activities against A549, MCF-7, and Hela cancer cell lines with IC_50_ values of 0.98 ± 0.08, 1.05 ± 0.17, and 1.28 ± 0.25 µM, respectively, as well as excellent kinase inhibitory activities (c-Met IC_50_ = 26.00 nM and VEGFR-2 IC_50_ = 2.6 µM). Moreover, compound **17l** inhibited the growth of A549 cells in G0/G1 phase in a dose-dependent manner, and induced the late apoptosis of A549 cells. Its intervention on intracellular c-Met signaling of A549 was verified by the result of Western blot. Fluorescence quantitative PCR showed that compound **17l** inhibited the growth of A549 cells by inhibiting the expression of c-Met and VEGFR-2, and its hemolytic toxicity was low. Molecular docking and molecular dynamics simulation indicated that compound **17l** could bind to c-Met and VEGFR-2 protein, which was similar to that of foretinib.

## 1 Introduction

Cancer is one of the leading causes of death, which seriously endangers human health. Traditional antitumor drugs have great toxicity and side effects because of lack in specificity, which cause serious damage to human tissues and organs (Maeda and Khatami, 2018) (Schirrmacher, 2019). In recent years, small molecular targeted antitumor drugs have been widely studied, whose high selectivity and specificity make the drug less toxic and with less side effects (Tarver, 2012) (Jemal et al., 2003). However, single target agents are more likely to develop drug resistance. Studies showed that small-molecule drugs interfering at the same time with multiple drug targets might be more effective to overcome drug resistance (Korcsmáros et al., 2007) (Giordano and Petrelli, 2008).

Mesenchymal–epithelial transition factor (c-Met) is a receptor in the family of receptor tyrosine kinases (RTKs) whose greatest characteristic is high-affinity for hepatocyte growth factor (HGF) and is involved in epithelial tissue remodeling and cell migration (Furge et al., 2000) (Moustakas and Heldin, 2007) (Alsahafi et al., 2019). c-Met kinase is located at the intersection of numerous tumor signaling pathways (Organ and Tsao, 2011), so dysregulation of the HGF/c-Met signaling pathway is a driving factor for many cancers, promoting tumor growth, invasion, spread, and/or angiogenesis (Parikh and Ghate, 2018), and is also associated with adverse clinical effects and drug resistance of some approved targeted therapies (Eder et al., 2009). Therefore, the development of novel and efficient small-molecule inhibitors targeting the HGF/c-Met signaling pathway has become a research hotspot (Liu et al., 2010). Vascular endothelial growth factor receptor (VEGFR) is a highly specific mitogen for vascular endothelial cells and is one of the most important regulators of angiogenesis activity (Ribatti, 2005). It can enhance vascular permeability, promote the growth, proliferation, migration, and differentiation of vascular endothelial cells, reduce cell apoptosis, induce vascular formation, change cytoskeleton function, and affect other cellular biological changes (Shibuya, 2001). Studies have shown that inhibition of VEGF signal cannot only block tumor angiogenesis but also change or destroy tumor blood vessels (Inai et al., 2004). Therefore, VEGFR is also an important target for tumor therapy. Several reports showed that dual c-Met/VEGFR inhibitors exhibited excellent antitumor activity and can also be able to compensate for the weakness of single-target inhibitors. Therefore, dual c-Met/VEGFR inhibitors may become a kind of potential antitumor agent, which can overcome drug resistance.

**FIGURE 1:**
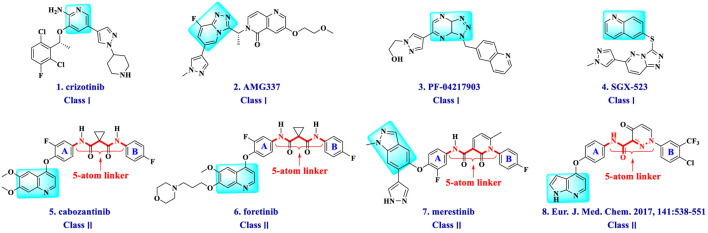
Structures of small-molecule mesenchymal–epithelial transition factor (c-Met) inhibitors.

Currently, lots of c-Met kinase inhibitors, such as crizotinib (Cui et al., 2011) (1, Class I, Figure 1) and cabozantinib (Fujiwara et al., 2004) (5, Class II), have been approved, respectively, for the treatment of non-small cell lung cancer and medullary thyroid cancer by the FDA. Moreover, enormous small-molecule c-Met inhibitors have been reported and entered clinical studies (Pasquini and Giaccone, 2018). These inhibitors can be classified into three types on the basis of the structural characters and the binding model to c-Met kinase: Class I, Class II, and the others (Zhang et al., 2020c). Class I c-Met inhibitors bind to ATP-binding sites to form a “U” shape, such as crizotinib (1), AMG337 (2) (Boezio et al., 2016), PF-04217903 (3) (Cui et al., 2012), and SGX-523 (4) (Buchanan et al., 2009). Class II c-Met inhibitors, which can bind multiple targets, usually occupy deep hydrophobic back pockets of ATP, such as cabozantinib (5) (Yakes et al., 2011), foretinib (6) (Zillhardt et al., 2011), merestinib (7) (Prins et al., 2016), and compounds (8) (Li et al., 2017). Moreover, foretinib and cabozantinib are also typical VEGFR-2 inhibitors.

**FIGURE 2:**
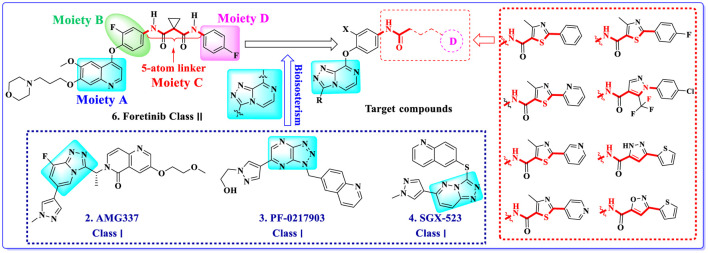
Design strategy and structures of target compounds.

Multitarget kinase inhibitors have achieved good therapeutic results in clinical trials, and antitumor therapy based on dual-target c-Met/VEGFR. Tyrosine kinase inhibitors have become one of the most effective clinical treatment methods for cancer. Therefore, we selected typical II c-Met inhibitor foretinib as the lead compound, introducing the active pharmacophore of VEGFR-2 inhibitors and modifying the five-atom portion, aiming to obtain a range of c-Met/VEGFR-2 dual inhibitors with good antitumor activity. As shown in Figure 2, the structure of type II c-Met inhibitor foretinib is divided into four parts, represented by A, B, C, and D. Moiety A, as the parent nucleus, is mostly an aromatic heterocyclic ring containing pyridine, such as quinoline and pyridine; moiety B is mostly substituted or unsubstituted phenoxy and pyridyloxy; moiety C can be a cyclic or chain structure, moiety B has six chemical bonds between moiety D, which is referred to as “five-atom regulation”; moiety D is a hydrophobic segment, mostly substituted phenyl group.

In this paper, we modified the following four moieties and designed a series of compounds with completely novel structures. First, the 4-phenoxy quinoline scaffold has been reported as an important component of VEGFR-2 targets, and the quinoline structure is also present in many c-Met inhibitors. Based on the principle of bioelectronic isosteric principle, triazolopyrazine was selected and introduced into foretinib to replace the quinoline structure. Triazolopyrazine is also the active pharmacodynamic structure of Class I c-Met inhibitors, which have good antitumor activity and pharmacokinetic parameters (Albrecht et al., 2008), and we also kept the phenoxy structure of moiety B. Based on the “five-atom regulation,” the five-membered heterocyclic rings with novel structure were introduced into part C by using the principle of fragmentation, such as pyrazole, iso-oxazole, and thiazole groups. The benzene ring in part D is used as a hydrophobic segment. In addition to introducing different types of substituents to adjust the electron distribution density on the benzene ring, it is also replaced with pyridine and thiophene structures to investigate these structures’ influence on the potency of the compounds. What is more, some other substituents were also introduced. The structural fragment of urea is still retained because it is the active pharmacodynamic fragment of VEGFR-2 inhibitors. As a result, a series of novel target compounds with triazolopyrazine structure were designed as dual c-Met/VEGFR-2 inhibitors (16a-l and 17a-m). Figure 2 illustrates the structures and the detailed design strategy of the target compounds.

The enzymatic inhibitory activity against c-Met kinase and the cellular effect on human lung adenocarcinoma (A549), human breast cancer (MCF-7), and human cervical carcinoma (Hela) cell lines of the synthesized compounds were assessed. The target compound **17l** showed good inhibitory effect on tumor cells and c-Met/VEGFR-2 kinase activity *in vitro.* Further studies on its apoptosis, cycle, and molecular docking were also conducted.

## 2 Materials and methods

### 2.1 Materials and Instruments

Reagents and materials used commonly were purchased commercially. We used the Büchi Melting Point B-540 instrument to measure melting points of target intermediates. The structure of compounds was confirmed by ^1^H NMR spectra, which were received by using a Bruker 400-MHz spectrometer and taking TMS as an internal standard, and mass spectra (MS) were recorded on electrospray ionization (ESI) mode on Agilent 1,100 liquid chromatography–mass spectrometry (LC-MS). Thin-layer chromatography (TLC) analysis, which was carried out on silica gel plates GF254 (Qingdao Haiyang Chemical, Qingdao, China), and the TLC plates were visualized by exposure to ultraviolet light (UV), was used to monitor the reaction process. All the reagents and materials were purchased commercially and utilized directly without purification except as otherwise noted.

### 2.2 Chemistry

**SCHEME 1:**
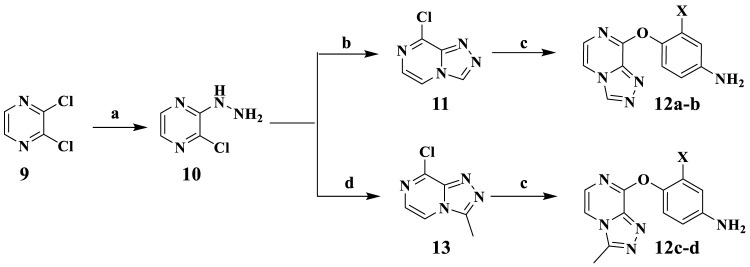
Reagents and conditions: **(A)** N_2_H_4_·H_2_O, EtOH, 85°C, reflux. **(B)** Triethoxy methane, 80°C, reflux. **(C)** 4-Aminophenol, KTB, KI, THF. **(D)** Triethyl orthoacetate, 80°C, reflux.

The synthetic procedure of intermediates **12a–b** and **12c–d** is illuminated in Scheme 1. Taking 2,3-dichloropyrazine (9) as the starting material, which is commercially available, the hydrazine group of compound **10** was substituted *via* nucleophilic reaction using hydrazine hydrate in the presence of compound **9** in ethanol. Compound **10** was cyclized with triethoxy methane to synthesize the key intermediate **11**. Then compounds **12a–b** were synthesized by substituting compound **11** with 4-aminophenol or 2-fluoro-4-aminophenol, respectively. The synthetic method of intermediates **12c–d** was consistent with that of compounds **12a–b**.

**SCHEME 2:**
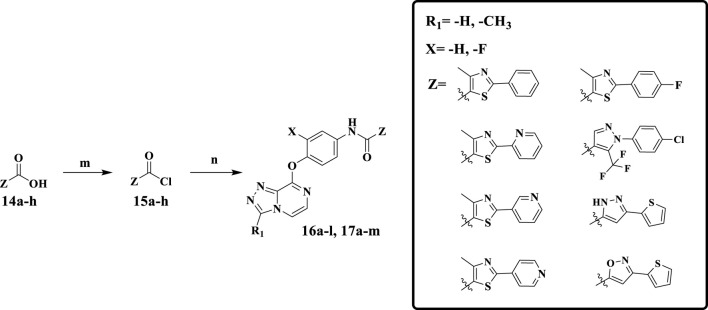
Reagents and conditions: (m) oxalyl chloride, DMF, CH_2_Cl_2_, r.t., 5 min; (*n*) DIPEA, CH_2_Cl_2_, r.t., 0.5 h.

Scheme 2 shows the synthetic route of target compounds **16a–l** and **17a–m**. Compounds **14a–h** reacted with oxalyl chloride to form intermediates **15a–h**. Finally, anilines **12a–b** and acyl chloride **15a–h**, catalyzed by DIPEA in dichloromethane at room temperature, formed the target compounds **16a–l**. The synthetic method of target compounds **17a–m** was the same as that of compounds **16a–l**.

### 2.3 Biological Evaluation

#### 2.3.1 Antitumor Assay

The standard MTT assay *in vitro* was used to evaluate the antitumor activities of target compounds (**16a-l** and **17a-m**) in A549, MCF-7, and Hela cell lines and foretinib was positive control. The cell lines used in the experiment were available from the Cell Culture Collection of Chinese Academy of Sciences (Shanghai Institutes for Biological Sciences, Chinese Academy of Sciences). Referring to the previous experimental methods of our research, all cells were cultured in suitable medium such as DMEM or RPMI-1640 containing 10% fetal bovine serum at 37°C in 5% CO_2_ and propagated for 24 h. Then the compounds with different gradient concentrations were added into the medium and incubated for 72 h, and then MTT (thiazolyl blue tetrazolium bromide) with a concentration of 5 µg/ml was added to every well for 4 h at 37°C. The formazan crystals were dissolved in 100 µl of DMSO in each well. The absorbancy at 492 nm (for absorbance of MTT formazan) and 630 nm (for the reference wavelength) was measured with an ELISA reader. All compounds were tested three times in each of the cell lines. The results, expressed as inhibition rates or IC_50_ (half-maximal inhibitory concentration), were the averages of two determinations and calculated by using the Bacus Laboratories Incorporated Slide Scanner (Bliss) software.

#### 2.3.2 Kinase Selectivity Assay

The target compounds were evaluated for their activity against c-Met kinase, taking foretinib as positive control. The kinase assay was performed by Pratzer Biotechnology Co. Ltd. (Changsha, China).

### 2.4 Cell Cycle Assay

The effect on cell cycle of the optimal compound **17l** was measured using foretinib as a control and performed by Pratzer Biotechnology Co. Ltd. (Changsha, China). A549 cells were placed in 12-well plates at a density of 10^6^/well with F-12 K containing 10% FBS as the medium, and the experiment was conducted on alternate days. Cells (1–5×10^6^) were collected, centrifuged at 1,500 rpm for 5 min to remove the supernatant, washed with PBS twice, and gently mixed with 200 μl of cell cycle rapid detection reagent to make a single-cell suspension, which was detected by flow cytometry within 1 h.

### 2.5 Cell Apoptosis Assay

We evaluated the effect of compound **17l** on cell apoptosis according to our previous methods (Zhao et al., 2020), using A549 cells, and taking foretinib as positive control. The cell apoptosis assay was performed by Pratzer Biotechnology Co. Ltd. (Changsha, China). After A549 cells were resuscitated, the cells were cultured in F-12 K medium containing 10% FBS and 1% pen/strep in incubator with 5% CO_2_ at 37°C. A549 cells, being in the logarithmic growth phase, were digested with 25% trypsin and laid in six-well plates with a density of 2 × 10^5^/well, and the cells were collected centrifugally after 72 h of treatment with each compound. The cells were washed twice with PBS, which was precooled to 4°C. Then the cells were suspended with 500 μl of buffer, and the concentration was adjusted to 10^6^/ml. A 100-μl suspension was placed in a 5-ml flow tube, adding 5 µl of Annexin V-APC, adding 5 μl of propidiμm iodide, and the mixture was mixed and incubated at room temperature without light for 15 min detecting cell apoptosis by flow cytometry (FACS).

### 2.6 Western Blot

Western blot was utilized to further analyze the effect of compound **17l** on the downstream signal protein of c-Met and VEGFR-2. The detailed operations refer to the previous method of our research group (Zhao et al., 2020). A549 cells were treated with different concentrations of compound 17l and foretinib (positive control) for 24 h, and then RIPA lysis buffer was utilized to lysate cells for whole-cell protein extraction. The extracted protein was transferred to a PVDF membrane in ice bath conditions after 10% SDS polyacrylamide gel electrophoresis separating the protein by size. The PVDF membrane was rinsed with phosphate-buffered solution (PBST), shaken in a Petri dish three times for 3–5 min every time, and incubated with different target proteins (p-c-Met, *p*-VEGFR-2, *p*-ERK 1/2, *p*-AKT, and GADPH). Finally, the film was exposed and developed in a darkroom. Image Lab software (Molecular Dynamics, Sunnyvale, CA, USA) was utilized to analyze the shadow density of each band, which was then expressed as a percentage of GDPDH band density (Zhao et al., 2020).

### 2.7 Fluorescence Quantitative PCR

Fluorescence quantitative PCR was conducted to analyze the expression patterns of c-Met and VEGFR-2 in the signal pathway in the adherent cells cultured with compounds **17a** and **17l**. The detailed operations refer to our previous published paper (He et al., 2019), and the primers used in the experiments are listed in Supplementary Table S1.

### 2.8 Hemolytic Test

The hemolytic toxicity of **17l** was studied with 1% Triton and 0.9% normal saline, and the former was a positive control, and the latter was a negative control. Triolatone (1%), 0.9% normal saline, and compound **17l** of different concentrations prepared in 0.9% normal saline reacted with the prepared sheep red blood cells, respectively. Photos were taken, and the absorbance value was measured by ultraviolet spectrophotometer at a wavelength of 540 nm, and hemolysis ratio (hemolysis %) was calculated according to the following formula (Guo et al., 2017). The experiment was repeated twice in parallel.

$$\mathrm{Hemolysis}\;\%=\frac{\mathrm{Abs}\left(\mathrm{experimental}\;\mathrm{group}\right)-\mathrm{Abs}\left(\mathrm{negative}\;\mathrm{control}\right)}{\mathrm{Abs}\left(\mathrm{positive}\;\mathrm{control}\right)-\mathrm{Abs}\left(\mathrm{negative}\;\mathrm{control}\right)}$$

### 2.9 Molecular Docking Study

The co-crystal structures of c-Met (PDB code 3LQ8) and VEGFR-2 (PDB code 4SAD) were selected as the template to generate docking models. The AutoDock 4.2 software (The Scripps Research Institute, United States ) was used to prepare the 3D structure of the docking ligands **17l** and perform energy minimization. c-Met protein as docking receptor went through the preparation process of flexible docking. Then compound **17l** was placed in the prepared system. After analyzing the interaction between **17l** and c-Met protein, the proper docking conformation was selected and preserved based on the calculated energy. PyMOL 1.8. x software (https://pymol.org) was utilized to perform and modify the docking results.

### 2.10 Molecular Dynamics Simulation

Gromacs 2019.3 (Lindahl et al., 2019) force field was used to deal with 3LQ8. For compound **17l**, restricted electrostatic potential (RESP) was calculated using Multiwfn (Wang et al., 2006) and eventually parameterized using Generation Amber Force Field (GAFF) (Wang et al., 2004). The long-range electrostatic interactions were calculated by the particle mesh Ewald (PME) (Darden et al., 1993). The prepared system had undergone double minimization. Compound **17l** was simulated for MD at 120 ns using the GROMACS-ver software. The 120-ns trajectory was analyzed after MD simulation, and the g-mmpbsa method was used to calculate the binding free energy and energy decomposition (Kumari et al., 2014).

## 3 Results and discussion

### 3.1 Chemistry

We synthesized all the compounds and intermediates by referring to our group’s previous research methods (Zhang B. et al., 2020) (Zhang et al., 2020b). ^1^H NMR and ESI-MS were utilized to characterize all the synthesized compounds and intermediates. The detailed operating steps and spectrum information were obtained in the Supplementary Material.

### 3.2 Biological Evaluation

The antiproliferative effects of the target compounds **16a–l** and **17a–m** had been evaluated toward three cancer cell lines A549, MCF-7, and Hela by the method of MTT [3-(4,5-dimethylthiazolyl-2)-2,5- diphenyltetrazolium bromide] with foretinib as positive control. Table 1 shows the results, and all the values were averages of two independent experiments. As shown from the chart, most of the compounds exhibited favorable antiproliferative activities against A549, MCF-7, and Hela cell lines, and most of them exhibited more excellent antiproliferative activities against A549 than that of MCF-7 and Hela. Among them, compound **17l** showed a wonderful antiproliferative activity against A549, MCF-7, and Hela cell lines, and their IC_50_ values were 0.98 ± 0.08, 1.05 ± 0.17, and 1.28 ± 0.25 µM, respectively, which was similar to that of the reference compound foretinib. Overall result of antiproliferative activity showed that the introduction of triazolo [4,3-*a*] pyrazine core could improve the antitumor effect of target compounds, which indicated that triazolo [4,3-*a*] pyrazine core was an active pharmacophore.

**TABLE 1:** Antiproliferative and mesenchymal–epithelial transition factor (c-Met) kinase inhibitory activities of the target compounds.

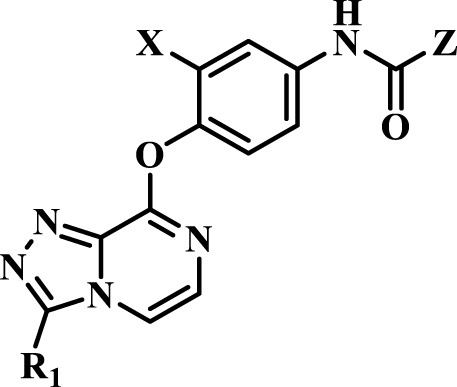							
Compounds	X	R_1_	Z	IC_50_ (μM)^a^			
				A549	MCF-7	Hela	c-Met
16a	H	H	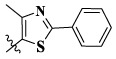	12.36 ± 0.97	13.76 ± 1.23	21.54 ± 1.78	0.794
16b	H	H	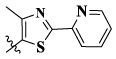	6.46 ± 0.33	7.87 ± 0.42	12.16 ± 1.08	0.379
16c	H	H	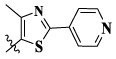	5.58 ± 0.75	6.45 ± 0.81	14.31 ± 1.15	0.345
16d	H	H	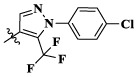	12.35 ± 0.98	11.52 ± 1.06	19.53 ± 1.94	0.722
16e	H	H	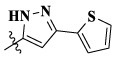	5.44 ± 0.42	7.13 ± 0.63	11.19 ± 0.96	0.342
16f	H	H	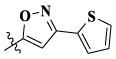	27.33 ± 0.86	>50	>50	>1
16 g	F	H	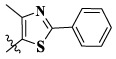	8.21 ± 0.65	9.17 ± 1.03	17.73 ± 1.29	0.658
16 h	F	H	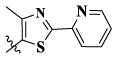	6.56 ± 0.42	8.18 ± 1.12	13.94 ± 1.59	0.437
16i	F	H	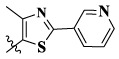	17.35 ± 0.97	19.03 ± 1.32	24.96 ± 2.17	ND^b^
16j	F	H	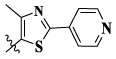	3.26 ± 0.67	3.68 ± 0.34	6.17 ± 0.58	0.110
16 k	F	H	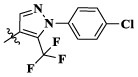	6.18 ± 0.34	6.51 ± 0.63	12.89 ± 1.06	0.380
16l	F	H	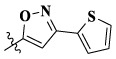	11.44 ± 0.76	11.97 ± 1.24	23.32 ± 2.12	0.725
17a	H	CH_3_	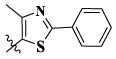	**1.23 ± 0.13**	**1.43 ± 0.21**	**2.31 ± 0.29**	**0.055**
17b	H	CH_3_	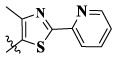	4.31 ± 0.49	5.15 ± 0.72	9.46 ± 0.58	0.245
17c	H	CH_3_	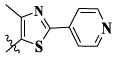	7.68 ± 0.71	8.79 ± 0.89	15.88 ± 1.10	0.532
17d	H	CH_3_	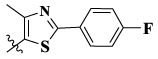	5.21 ± 0.37	6.95 ± 0.67	10.73 ± 1.42	0.289
17e	H	CH_3_	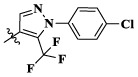	**2.05 ± 0.19**	**1.74 ± 0.22**	**4.78 ± 0.43**	**0.077**
17f	H	CH_3_	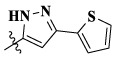	5.73 ± 0.54	6.38 ± 0.85	12.37 ± 1.53	0.300
17 g	F	CH_3_	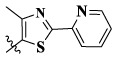	4.65 ± 0.66	4.83 ± 0.33	9.25 ± 0.45	0.232
17 h	F	CH_3_	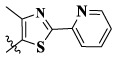	3.56 ± 0.32	4.25 ± 0.76	7.43 ± 0.38	0.156
17i	F	CH_3_	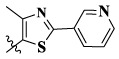	188	25.44 ± 1.54	>50	ND
17j	F	CH_3_	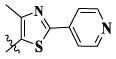	7.16 ± 0.45	7.31 ± 0.77	14.18 ± 1.64	0.450
17 k	F	CH_3_	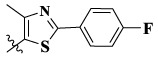	15.34 ± 0.51	18.17 ± 1.28	25.33 ± 2.40	ND
17l	F	CH_3_	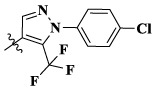	**0.98 ± 0.08**	**1.05 ± 0.17**	**1.28 ± 0.25**	**0.026**
17 m	F	CH_3_	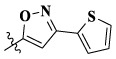	8.84 ± 0.97	9.34 ± 1.03	17.37 ± 1.56	0.653
Foretinib^c^	-	-	-	0.71 ± 0.05	0.93 ± 0.09	1.18 ± 0.25	0.019

Note. A549, human lung adenocarcinoma; MCF-7, human breast cancer; Hela, human cervical carcinoma.

a The values are the average of two separate determinations.

b ND, not detected.

c Used as a positive control.

Moreover, compared with compounds **16a–f** (X = H) without a substituent, the introduction of an F atom on the phenoxy group in compound **16g–l** is favorable for antiproliferative activity. The same trend had also been observed between compounds **17a–f** and **17g–m**. By comparing with compounds **16a–l** and **17a–m**, it was found that compounds with methyl substitution (R_1_ = H) had higher antiproliferative activity against three tumor cell lines. Comparing compounds **17a** and **17d** (or **17g** and **17k**), compound **17a** without an F atom substitution on the benzene ring had higher antiproliferative activity against the three tumor cell lines. Compounds **17b–17c** and **17h–17j** showed that the introduction of the 2-pyridine group into the 5-methylthiazole ring had better antiproliferative activity on the three kinds of tumor cells. Compounds **17e** and **17l** showed better antiproliferative activity and kinase activity when the five-atom part was 5-(trifluoromethyl)-1*H*-pyrazole.

Furthermore, taking foretinib as positive control, we tested the c-Met inhibitory activities of target compounds. Most target compounds exhibited moderate inhibitory activities of c-Met kinase according to experimental results (Table 1). Specifically, compounds **17a**, **17e**, and **17l** showed excellent activity against c-Met kinase at the nanomolar level, with the IC_50_ values of 55, 77, and 26 nM, respectively. It is worth noting that the activity against c-Met kinase of compound **17l** was equal to positive control foretinib (19.00 nM). The most active compound **17l** was selected to further test the inhibitory activities against VEGFR-2 and EGFR^wt^ kinases, and also test the antiproliferative activity against three cell lines using sorafenib (multitarget inhibitors through inhibiting VEGFR and PDGFR) as positive control, including human non-small cell lung cancer cells (H460), human lung adenocarcinoma cell line harboring a deletion in exon 19 of EGFR (PC-9), and non-small-cell lung cancer cells harboring T790M-targeted epidermal growth factor receptor mutation to the tyrosine kinase inhibitor gefitinib cell line (H1975). The results indicated that the inhibitory activity of compound **17l** against H1975 and H460 cells was similar with positive control, while the antiproliferative activity against PC-9 cells was worse than it. The inhibitory activities (Table 2) demonstrated that **17l** was a potential dual c-Met/VEGFR-2 inhibitor with high selectivity to EGFR^wt^.

**TABLE 2:** Antiproliferative and some other kinase inhibitory activities of compound **17l.**

Compound	IC_50_ (μM)					
	c-Met	VEGFR-2	EGFR^WT^	PC-9	H460	H1975
17l	0.026	2.6	>10	38.25 ± 1.58	5.86 ± 0.77	15.30 ± 1.18
Staurosporine^a^	0.125	-	-	ND^c^	ND	ND
Sorafenib^b^	ND	ND	ND	5.41 ± 0.73	5.27 ± 0.72	10.18 ± 1.00

Note. VEGFR, vascular endothelial growth factor receptor; PC-9, exon 19 of EGFR; H1975, gefitinib cell line; H460, human non-small cell lung cancer cells.

a Used as a positive control.

b Used as a positive control.

c ND, not detected.

**FIGURE 3:**
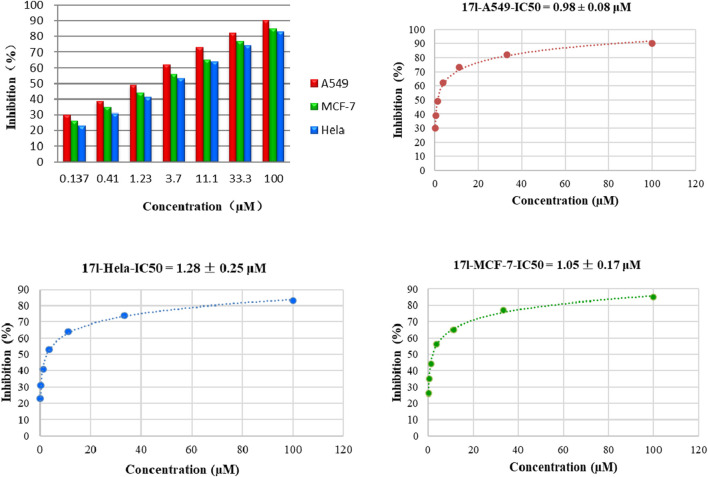
Relationship between the inhibition rate and concentration of compound **17l** on three cancer cell lines.

Additionally, we also selected the optimal compound **17l** to explore the relationship between antiproliferative activity and compound concentration. Three cell lines (A549, MCF-7, and Hela) were treated with compound **17l** at different concentrations for 72 h. Figure 3 illustrates that with the increase in compound **17l** concentration, its inhibitory ratio against the three cell lines increased gradually, presenting dose dependency. Furthermore, the antiproliferative activity of compound **17l** on A549 cells was better than that on MCF-7 and Hela cells.

### 3.3 Effect on Cell Cycle Progression

**FIGURE 4:**
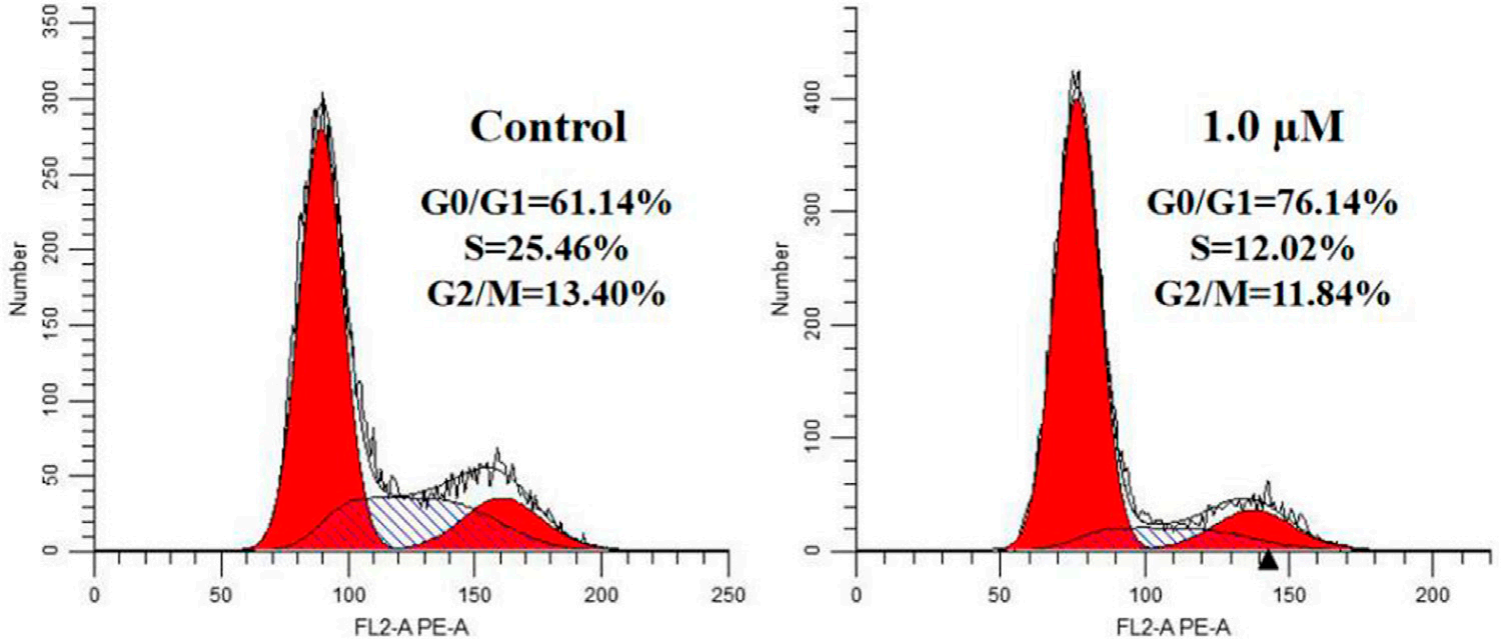
Cell cycle progression analyses of human lung adenocarcinoma (A549) cells cultured with compound **17l** for 72 h.

We treated A549 cells with 1.0 μM compound **17l** for 72 h and determined its effect on cell cycle by flow cytometry to further reveal the growth inhibition mechanism of the target compound on tumor cells. The results are shown in Figure 4. Compared with the control group, A549 cells cultured with compound **17l** had a significant inhibitory effect on cell growth in G0/G1 phase, and the cell block rate increased from 61.14% to 76.14%. At the same time, the content of cell block rate decreased in S phase, but the change in G2/M phase was not obvious. In conclusion, compound **17l** could inhibit the growth of A549 cells in the G0/G1 phase.

### 3.4 Effect on Apoptosis

**FIGURE 5:**
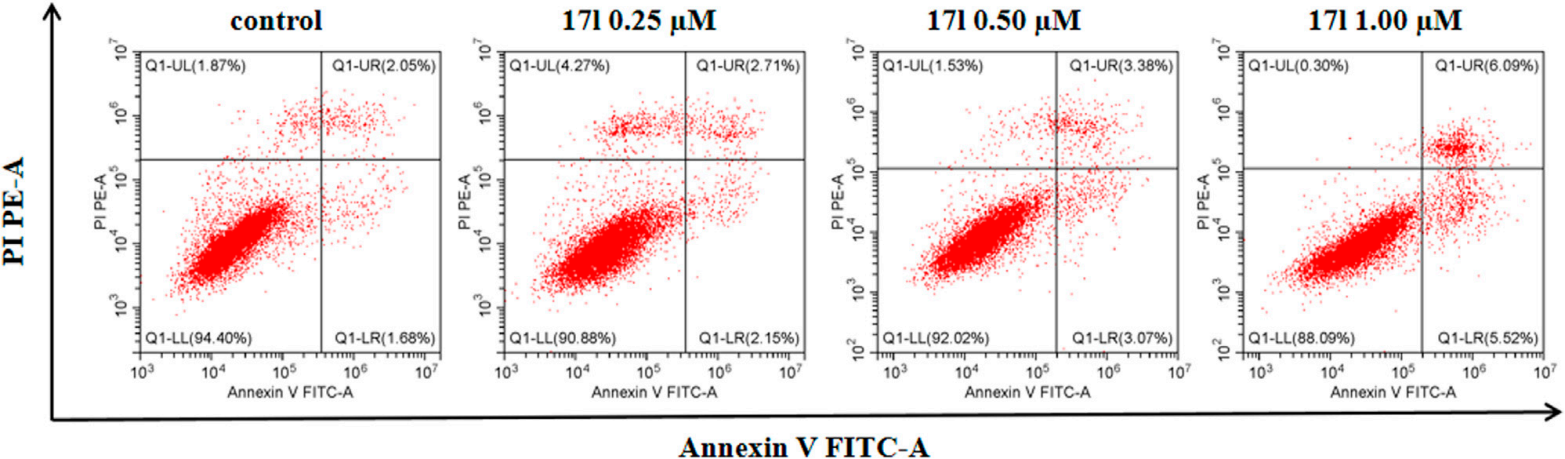
The analyses of compound **17l** induces apoptosis of A549 cells.

The assay of annexin V/PI staining was performed to deeper research the mechanism of apoptosis induced by compound **17l** in A549 cells. Figure 5 shows the analysis results of A549 cells cultured with compound **17l** for 72 h. In comparison with the control group (the total apoptosis rate was 3.73%), A549 cells were treated with 0.25, 0.50, and 1.00 μM, the total apoptosis rates of compound **17l** were 4.86%, 6.45%, and 11.61%, respectively. In conclusion, compound **17l** induced apoptosis of A549 cells in a dose-dependent manner, and the effect of late apoptosis was obvious.

### 3.5 Modulation of C-Met Activity in A549 Cell Line by Compound 17l

**FIGURE 6:**
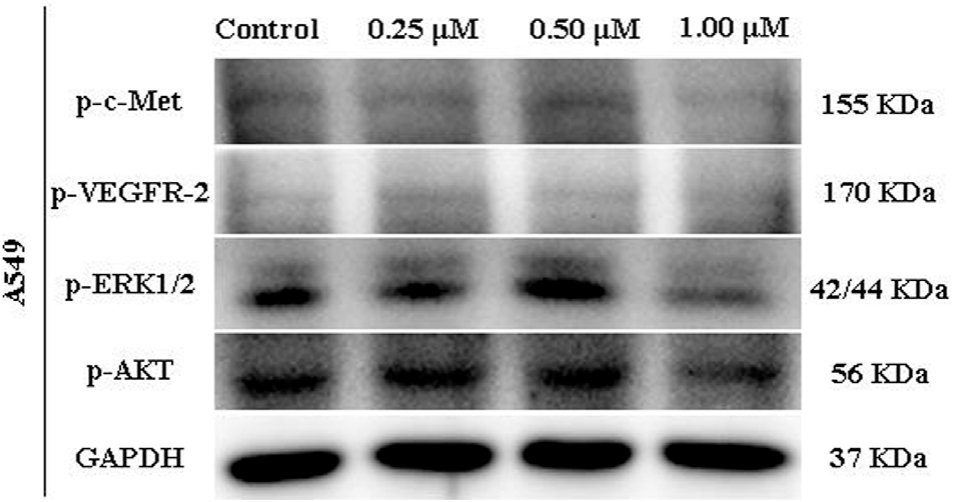
c-Met biomarker modulation by compound **17l** (Evaluated by Western blot).

We further verified the effect of compound **17l** on c-Met and downstream signaling pathways. We treated A549 cell line with different doses of compound **17l** for 24 h to measure its phosphorylation inhibition in different pathways. At different concentrations, compound **17l** inhibited the protein levels of p-c-Met (Tyr1003) (Figure 6) and *p*-VEGFR-2 (Tyr1059) (Figure 6), indicating that it can inhibit phosphorylation protein of c-Met and VEGFR-2, but the inhibitory effect on *p*-VEGFR-2 is not obvious. At the same time, we observed that at a concentration of 1.00 μM, compound **17l** significantly inhibited the phosphorylation protein of c-Met’s downstream biomarker signals ERK1/2 and AKT. This result further indicates that compound **17l** may be an effective c-Met inhibitor.

### 3.6 Fluorescence Quantitative PCR Analysis

**FIGURE 7:**
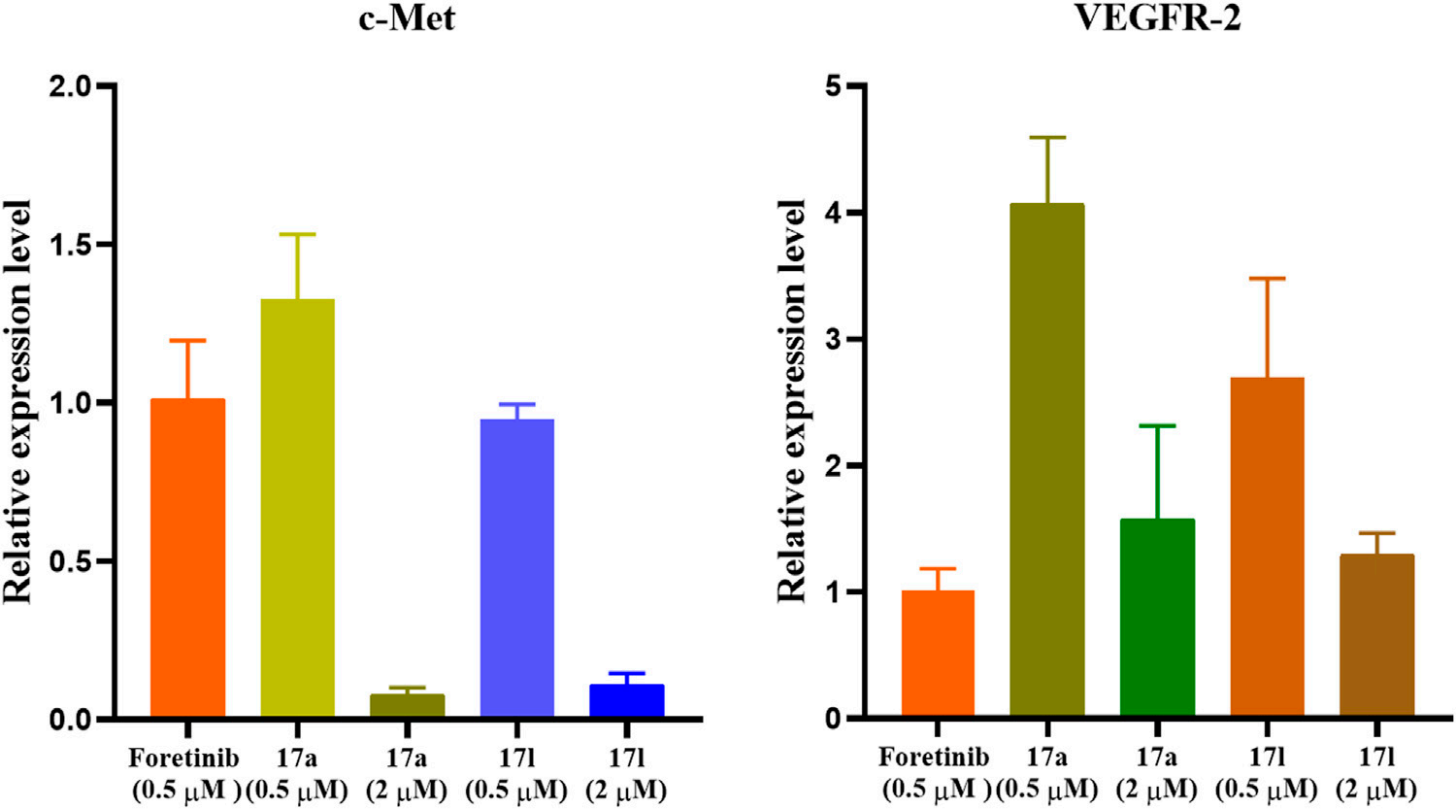
Gene expression pattern of c-Met and vascular endothelial growth factor receptor (VEGFR)-2 in A549 cells treated with foretinib, **17a**, and **17l**.

Different amounts of apoptosis cells resulted in different gene expression levels. Therefore, compounds **17a** and **17l** with the best antiproliferation activity were selected as representatives to investigate c-Met/VEGFR-2 gene expression level in tumor cells for further verifying the results of anti-proliferation activity. Compounds **17a** and **17l** with the best antiproliferative activity were selected as representatives. The expression patterns of c-Met and VEGFR-2 kinases in the signal pathway were analyzed by fluorescent quantitative PCR. In the results shown in Figure 7, the expression of c-Met gene in A549 cells treated with compound **17a** was higher than that of foretinib at 0.5 μM, while the expression of c-Met gene in A549 cells treated with compound **17l** was comparable with foretinib. However, VEGFR-2 gene expression was higher in both **17a-** and **17l**-treated A549 cells than in foretinib cells at 2 μM. The results were consistent with the antiproliferative activity of our compounds.

### 3.7 Hemolytic Test

**FIGURE 8:**
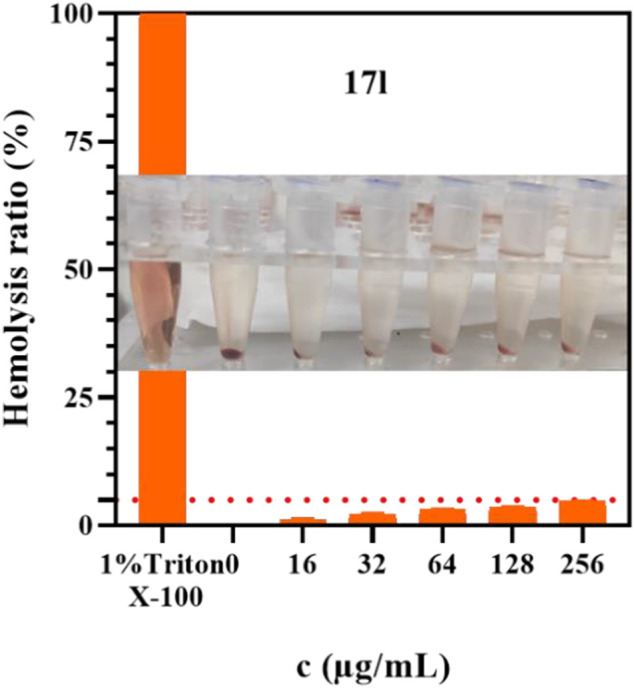
The hemolysis rate of compound **17l** with different concentrations.

The best compound **17l** with antiproliferative activity and kinase inhibition activity was selected to detect its hemolytic toxicity at a gradient concentration of 16–256 μg/ml, and the safety of intravenous administration was preliminarily investigated. In the results shown in Figure 8, the hemolytic rate of compound **17l** was still less than 5% even at the highest concentration within the study range, which proved that compound **17l** had low hemolytic toxicity to red blood cells. Its hemolytic toxicity meets the requirements of intravenous administration, which provides a basis for further *in vivo* research.

### 3.8 Molecular Docking Study

**FIGURE 9:**
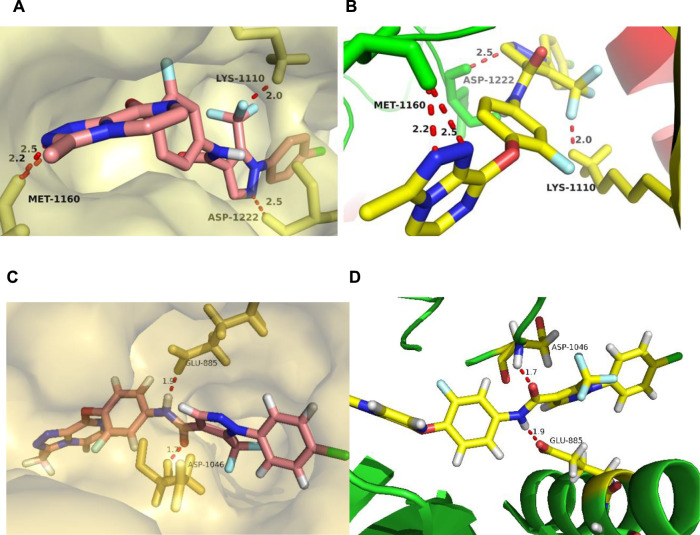
The docking mode of compound **17l** with c-Met (PDB code 3LQ8) and VEGFR-2 (PDB code 4ASD). **(A,B)** showed three major hydrogen bond interactions formed between the **17l** and c-Met protein. **(C,D)** showed two hydrogen bond interactions between the **17l** and VEGFR-2 protein.

Taking comprehensive consideration of c-Met and VEGFR-2 inhibitory activities and antiproliferative activities, compound **17l** was chosen as a representative in exploring the binding model of target compounds with c-Met and VEGFR-2 protein. Compound **17l** was docked with c-Met protein (PDB code 3LQ8) and VEGFR-2 protein (PDB code 4SAD), respectively, which used the AutoDock 4.2 software (The Scripps Research Institute, USA), and the PyMOL 1.8 x software (https://pymol.org) was used to modify the docking results. The entire heterocyclic skeleton of compound **17l** was closely embedded into the hydrophobic cavity of c-Met protein in Figure 9A and as shown in Figure 9C, the compound was also tightly embedded in the hydrophobic cavity of VEGFR-2 protein. In addition, there were two N atoms on the triazolopyrazine ring of compound **17l**, which formed a bidentate hydrogen bond with MET-1160 that was the key amino acid residue in the hinge region of the protein. The result indicated that the introduction of triazolopyrazine ring was the key to guarantee the activity of target compounds (Figure 9B). Meanwhile, the oxygen atom of the pyridazinone group of the five-atom linker formed a hydrogen bond with LYS-1110, and the two nitrogen atoms on the pyridazinone group constituted a bidentate hydrogen bond with residue ASP-1222, which also indicated that the target compounds had strong binding ability with c-Met protein. In Figure 9D, the result indicated that the amide fragment of the five-atom linker of **17l** formed hydrogen bonds with GLU-885 and ASP-1046 of VEGFR-2 kinase, respectively, improving the binding ability of the compound to the kinase to a certain extent. The results of molecular docking illustrated that compound **17l** might be a potential dual inhibitor of c-Met/VEGFR-2.

### 3.9 Molecular Dynamic Simulation

**FIGURE 10:**
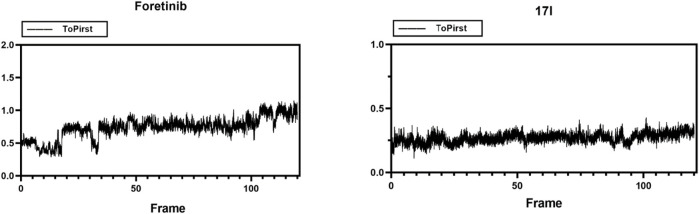
Root-mean-squared deviation (RMSD) plots for the backbone atoms and foretinib or **17l** during 120-ns MD simulations. The *X* axis is the frame number. The *Y* axis is the RMSD value.

Molecular dynamic simulations were performed in Gromacs 2019.3 in order to assess the stability of a system of protein with small molecules. Compound **17l** and c-Met protein (3LQ8) systems were selected as representatives for the simulation, and the whole process lasted for 120 ns. MD simulations trajectories were utilized for data extracting and binding free energy calculating. As shown in Figure 10, the root-mean-squared deviation (RMSD) values, of the protease backbone atoms relative to their crystal structures during the MD simulation, indicated that the ligand–receptor system of foretinib-3LQ8 as control tended to be stable at 3.5 ns and the distribution of the **17l**–3LQ8 system also tended to be stable quickly. The results indicated that the intramolecular system was stable, and the dynamic simulation was meaningful.

The MM-PBSA method was utilized to calculate the total binding free energy of **17l**, which were −58.850 ± 0.765, −9.239 ± 0.510, 39.615 ± 0.624, −5.470 ± 0.058, and −33.944 ± 0.961 kcal/mol for the complexes of **17l**–3LQ8, respectively (Table 3). The results show that the total free energy of compound **17l** was close to that of foretinib. As the docking studies demonstrated, the energies of van der Waal energy (−58.850 ± 0.765 kcal/mol) and binding energy (−33.944 ± 0.961 kcal/mol) of compound **17l** were lower than those of foretinib, which may interpret its higher binding free energy.

**TABLE 3:** Predicted free energies (kcal/mol) for binding of foretinib and **17l** to c-Met kinase.

Energy terms (kcal/mol)	Foretinib	17l
VDW energy^a^	−56.890 ± 1.764	−58.850 ± 0.765
EE energy^b^	−10.453 ± 1.081	−9.239 ± 0.510
Polar energy^c^	41.902 ± 2.074	39.615 ± 0.624
Apolar energy^d^	−6.008 ± 0.132	−5.470 ± 0.058
Delta total^e^	−31.448 ± 1.414	−33.944 ± 0.961

Note. a Van der Waal energy.

b Electrostattic energy.

c Polar solvation energy.

d SASA, energy.

e Binding energy.

**FIGURE 11:**
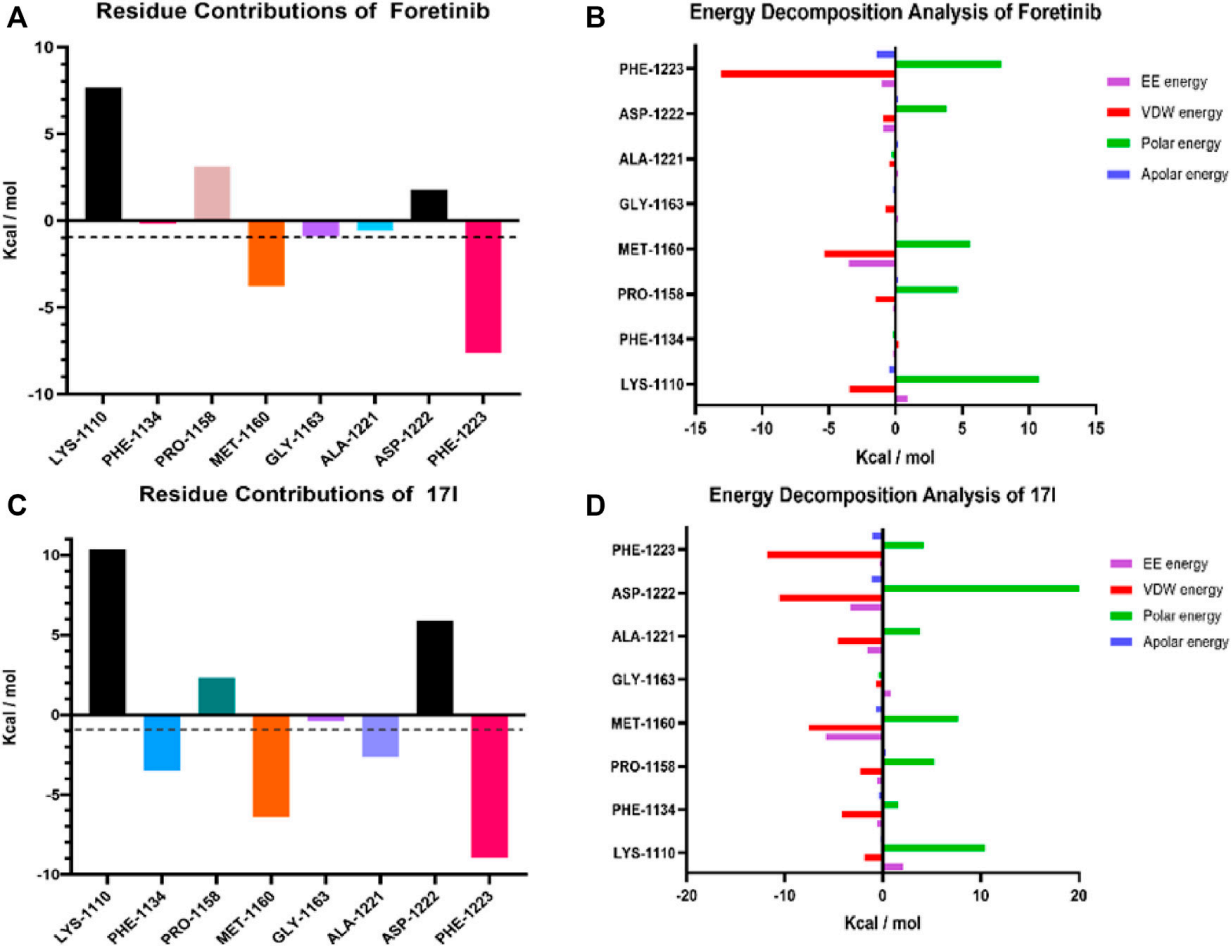
Molecule dynamic stimulation results of foretinib-3LQ8 and **17l**–3LQ8. Residue contributions of potential hot residues of **(A)** and **(C)**. Energy decomposition of potential hot residues of **(B)** and **(D)**. The unit of energy is kcal/mol.

The g_mmpbsa method was used to calculate residue contributions of potential hot residues of foretinib and compound **17l** in order to evaluate the contribution of residues in the binding model of c-Met protein. In addition, to explore dominated interactions in the ligand–protein system, energy decomposition of potential hot residues was performed. For foretinib (Figure 11A), the results demonstrated that the residues may be significant for inhibitory effect: MET-1160 (−3.771 kcal/mol) and PHE-1223 (−7.607 kcal/mol). For compound **17l** (Figure 11C **)**, the results suggest that PHE-1134 (−3.5073 kcal/mol), MET-1160 (−6.3982 kcal/mol), ALA-1221 (−2.6418 kcal/mol), and PHE-1,223 (−8.9509 kcal/mol) may be important residues. Among these residues, MET-1160 and PHE-1223 was the most desirable for the total binding free energies, and the interaction with LYS-1,110, PRO-1,158, and ASP-1,222 seemed like unfavorable. The results show that the docking result of compound **17l** was better than that of foretinib. The binding of ligand–receptor by EE energy and VDW energy was necessary (Figures 11B, D).

## 4 Conclusion

This study reports the synthesis and antiproliferative activities of [1,2,4]triazolo [4,3-a]pyrazine derivatives **16a–17m**. Most derivatives performed moderate to significant potency, and the most promising compound **17l** (c-Met kinase IC_50_ = 0.026 µM) exhibited excellent antiproliferative activities against A549, MCF-7, and Hela cancer cell lines with IC_50_ values of 0.98 ± 0.08, 1.05 ± 0.17, and 1.28 ± 0.25 µM, respectively. Compound **17l** could arrest A549 cell cycle in the G0/G1 phase and induce apoptosis in A549 cells in a dose-dependent manner. The docking study indicated that the introduction of 4-methyl-5-(trifluoromethyl)-1*H*-pyrazole as the five-atom linker between moiety A and moiety B maintained the potent cytotoxicity. Therefore, compound **17l** may be a potential dual c-Met/VEGFR-2 kinase inhibitor, which can be further studied in the hope of producing more effective anticancer drugs.

## Data Availability

The raw data supporting the conclusion of this article will be made available by the authors, without undue reservation.
