# Supplementary material for: Design, Synthesis, and Biological Evaluation of [1,2,4]triazolo[4,3-a] Pyrazine Derivatives as Novel Dual c-Met/VEGFR-2 Inhibitors
Source: Front Chem. 2022 Apr 6;10:815534. doi: 10.3389/fchem.2022.815534 (PMC9019572; doi:10.3389/fchem.2022.815534)
Supplement: Supplementary file 1 [file DataSheet1.PDF]

Supplementary Material

Design, synthesis and biological evaluation of [1,2,4]triazolo[4,3-*a*] pyrazine derivatives as novel dual c-Met/VEGFR-2 inhibitors

Xiaobo Liu[1,3,†], Yuzhen Li[1†], Qian Zhang[1], Qingshan Pan[1], Pengwu Zheng[1], Xinyang Dai[1], Zhaoshi Bai[2*],Wufu Zhu[1*].

[1] Jiangxi Provincial Key Laboratory of Drug Design and Evaluation, School of Pharmacy, Jiangxi Science & Technology Normal University, Nanchang, China. [2] Jiangsu Cancer Hospital & Jiangsu Institute of Cancer Research & the Affiliated Cancer Hospital of Nanjing Medical University, Nanjing, China. [3] School of Chemical Engineering, Dalian University of Technology, Dalian, China.

†These authors have contributed equally to this work and share first authorship.

* Correspondence:

Zhaoshi Bai,

Baizhaoshi23@126.com

Wufu Zhu

zhuwufu-1122@163.com

Table S1 Real-time fluorescence quantitative primers.

Primers	Genetic Sequence
MET-qF	CCACGGGACAACACAATACA
MET-qR	TAAAGTGCCACCAGCCATAG
VEGFR-qF	AGCAGGATGGCAAAGACTAC
VEGFR-qR	TACTTCCTCCTCCTCCATACAG
GADPH-qF	GACAACATCCAGGGTATCACTAAGC
GADPH-qR	GGTCTCCTCGTAGATCATGGCA

Operating process and spectrum information

1. General Information

Reagents and materials used commonly were purchased commercially. We used Büchi Melting Point B-540 instrument to measure melting points of target intermediates. The structures of all compounds were confirmed by ^{1}H NMR spectra and Mass spectra (MS). The former data were received by using a Bruker 400 MHz spectrometers and taking TMS as an internal standard, and the latter were recorded on electrospray ionization (ESI) mode on Agilent 1100 Liquid chromatography–mass spectrometry (LC-MS). Thin layer chromatography (TLC) analysis was used to monitor the reaction process, which was carried out on silica gel plates GF254 (Qingdao Haiyang Chemical, Qingdao, China) and the TLC plates were visualized by exposure to ultra violet light (UV). The whole reagents and materials were purchased commercially and utilized directly without purification except as otherwise noted.

2. Detailed procedure for the preparation of key immediates and target compounds.

2.1 Synthetic steps of intermediate **10**

Taking 2,3-dichloropyrazine (**9**, 20 g, 0.134 mol) as starting material, a little amount of hydrazine hydrate was added for several times to reflux at 85℃, and the reaction was detected by TLC until the reaction was complete. After cooling, the reaction liquid was added to ice water and stirred. Precipitation occurred in the system and was filtered. The solid separated out was dried to obtain 16.8 g yellow powder (**10**), and the yield was 84.1%.

2.2 Synthetic steps of intermediate **11**

10 was added to triethyl orthoformate and reacted at 80°C, with TLC monitoring the reaction process. The reaction liquid was cooled and filtered, and petroleum ether was used to wash obtained solid. Finally, 4.8 g yellow solid (**11**) was obtained and the yield was 90.1%.

2.3 Synthetic steps of intermediates **12a-d**

P-aminophenol and potassium tert-butanol were added into the flask and stirred in ice bath for one hour with tetrahydrofuran as the solvent under the protection of nitrogen. The intermediate **11** (1 g, 6.5 mmol) and potassium iodide (0.12 g, 0.72 mmol) were added to the flask, and tetrahydrofuran was used as the solvent. The reaction system was heated to 80 °C and stirred. The reaction process was monitored by TLC under the protection of nitrogen. After the reaction was completed, the filtrate was drained, and a little amount of sodium hydroxide aqueous solution was added to make the system precipitation. The filter cake was dried, and 0.45 g gray solid **12a** was obtained, and the yield was 30.6%.

2.3 Synthetic steps of side chains **15a-h**

At room temperature, compound **14a-h** was added to 10 mL dichloromethane. Then DMF and appropriate oxaloyl chloride were added into the system and the reaction was monitored by TLC. The solution can be used for the next step without further purification.

2.4 Synthetic steps of compounds **16a-l** and **17a-m**.

15a-h was added drop-by-drop to the system of dichloromethane (10 mL) with **12a-d** (0.51 mmol) and N, N-diisopropylethylamine (0.49 mmol) in ice bath. Then the reaction system was stirred at room temperature for 30 min and monitored with TLC. The reaction solution was washed with 10% K_2CO_3 solution (50 mL) for three times and sodium chloride solution (50 mL) for once. The organic phase was dried by anhydrous sodium sulfate and concentrated, and the crude product was purified by silica gel column to obtain pure compounds **16a-l** and **17a-m**.

3. Spectrum information

N-(4-([1,2,4]triazolo[4,3-*a*]pyrazin-8-yloxy)phenyl)-4-methyl-2-phenylthiazole-5-carboxamide (16a)

Yellow solid in 31.5%, M.P.: 265.8–268.1 °C. ^{1}H NMR (400 MHz, DMSO-d_6) δ 10.41 (s, 1H), 9.48 (d, J = 4.3 Hz, 1H), 8.32 (d, J = 5.0 Hz, 1H), 8.01 (s, 2H), 7.79 (d, J = 8.6 Hz, 2H), 7.56 (s, 3H), 7.39–7.32 (m, 3H), 2.68 (s, 3H). TOF MS ES+ (*m/z*): [M + H]$^+$, calcd for $C_{22}H_{16}N_6O_2S$: 429.1134, found, 429.1055.

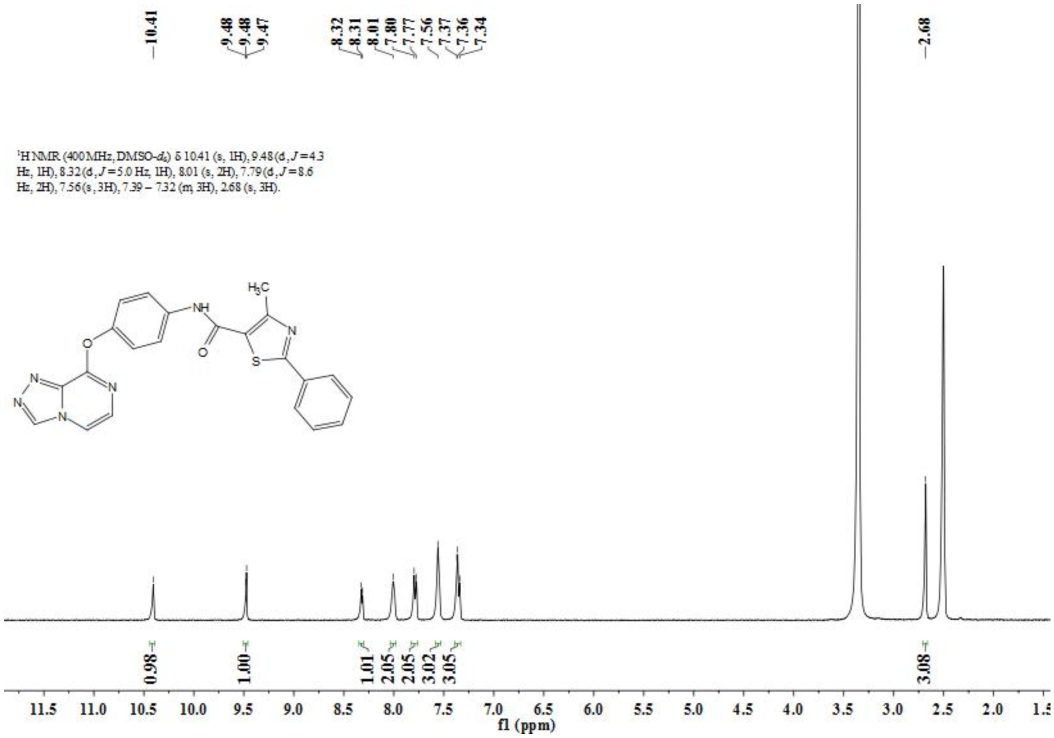

Figure 1. ^{1}H NMR spectra of compound **16a.**

N-(4-([1,2,4]triazolo[4,3-*a*]pyrazin-8-yloxy)phenyl)-4-methyl-2-(pyridin-2-yl)thiazole-5-carboxamide (16b)

Yellow solid in 54.1%, M.P.: 270.0–273.2 °C. ^{1}H NMR (400 MHz, DMSO-d_6) δ 10.44 (s, 1H), 9.48 (s, 1H), 8.68 (d, *J* = 4.3 Hz, 1H), 8.31 (d, *J* = 4.7 Hz, 1H), 8.18 (d, *J* = 7.9 Hz, 1H), 8.01 (t, *J* = 7.5 Hz, 1H), 7.80 (d, *J* = 7.7 Hz, 2H), 7.60–7.52 (m, 1H), 7.35 (d, *J* = 7.1 Hz, 3H), 2.69 (s, 3H). TOF MS ES+ (*m/z*): [M + H]$^+$, calcd for $C_{21}H_{15}N_7O_2S$: 430.1086, found, 429.9987.

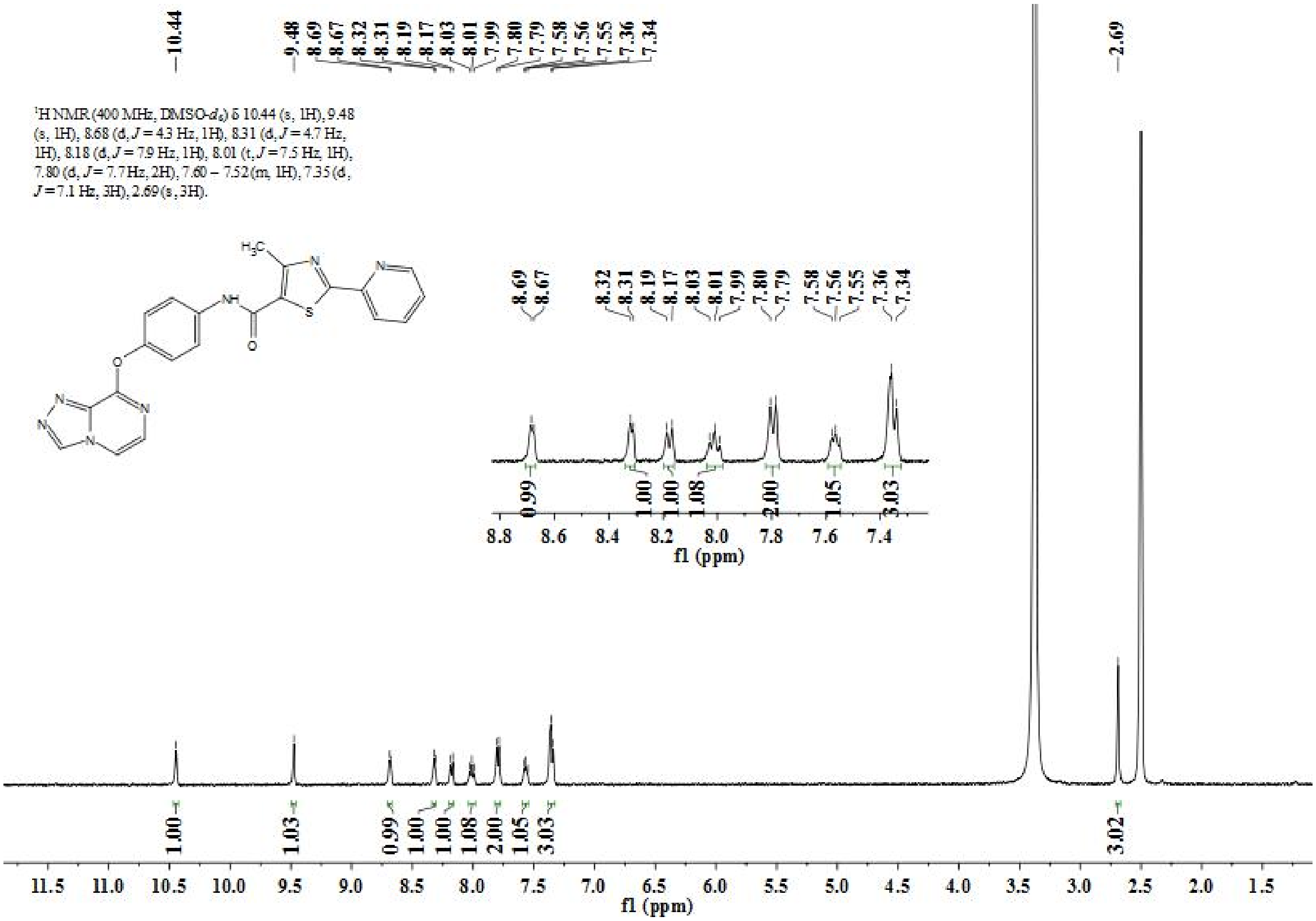

Figure 2. ^{1}H NMR spectra of compound **16b.**

N-(4-([1,2,4]triazolo[4,3-*a*]pyrazin-8-yloxy)phenyl)-4-methyl-2-(pyridin-4-yl)thiazole-5-carboxamide (16c)

Yellow solid in 41.5%, M.P.: 235.6–237.5 °C. ^{1}H NMR (400 MHz, DMSO-d_6) δ 10.53 (s, 1H), 9.47 (s, 1H), 8.76 (s, 2H), 8.32 (s, 1H), 7.94 (s, 2H), 7.79 (d, *J* = 8.7 Hz, 2H), 7.37 (s, 3H), 2.70 (s, 3H). TOF MS ES+ (*m/z*): [M + H]$^+$, calcd for $C_{21}H_{15}N_7O_2S$: 430.1086, found, 430.1008.

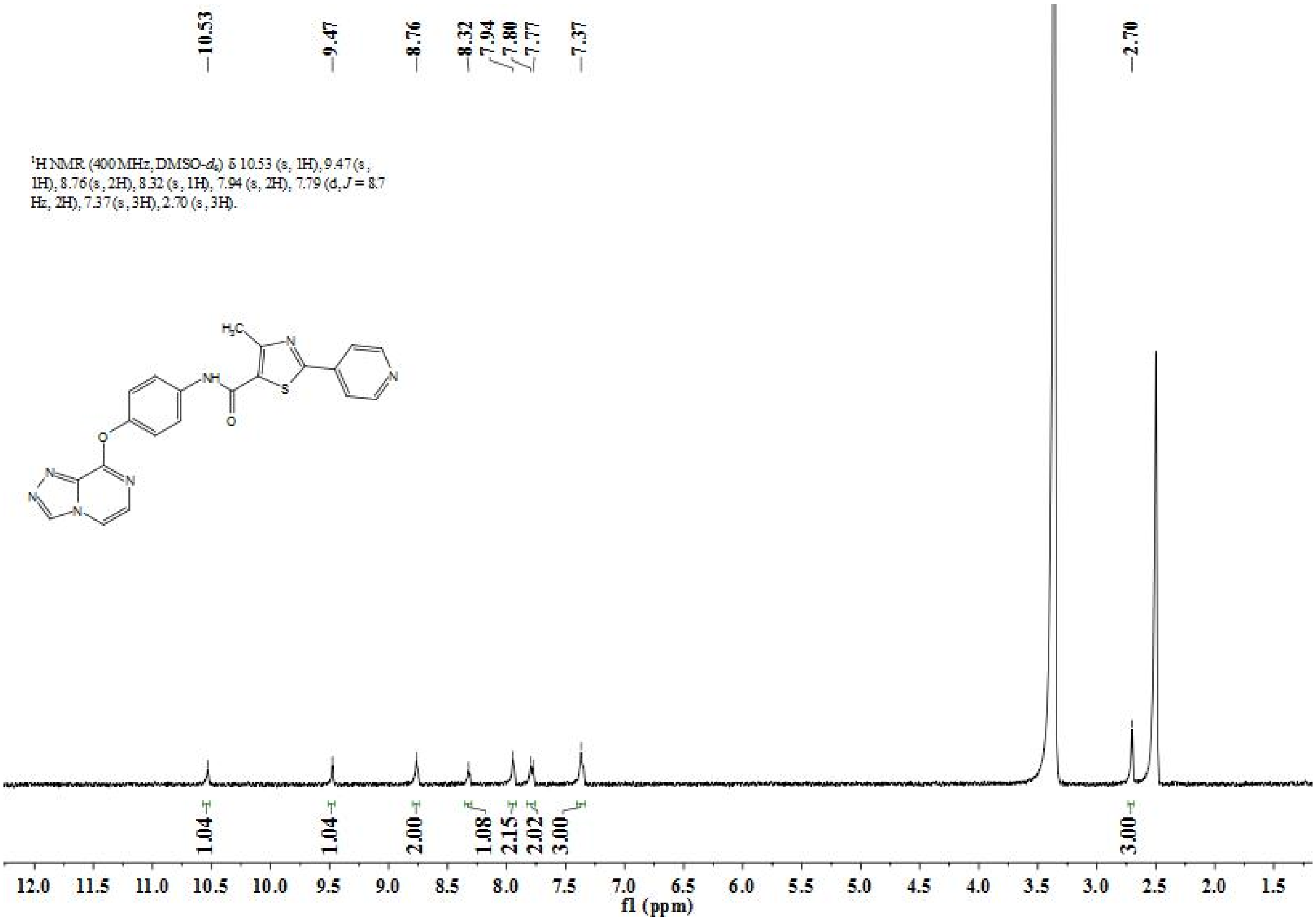

Figure 3. ^{1}H NMR spectra of compound **16c.**

N-(4-([1,2,4]triazolo[4,3-*a*]pyrazin-8-yloxy)phenyl)-1-(4-chlorophenyl)-5-(trifluoromethyl)-1H-pyrazole-4-carboxamide (16d)

Light yellow solid in 20.2%, M.P.: 261.3–263.5 °C. ^{1}H NMR (400 MHz, DMSO-d_6) δ 10.76 (s, 1H), 9.42 (d, J = 2.1 Hz, 1H), 8.29 (s, 2H), 7.80 (d, J = 12.4 Hz, 1H), 7.61 (t, J = 4.8 Hz, 2H), 7.49 (dd, J = 31.2, 9.2 Hz, 5H), 7.31 (s, 1H). TOF MS ES+ (m/z): [M + H]$^+$, calcd for $C_{22}H_{13}ClF_3N_7O_2$: 500.0850, found, 500.0971.

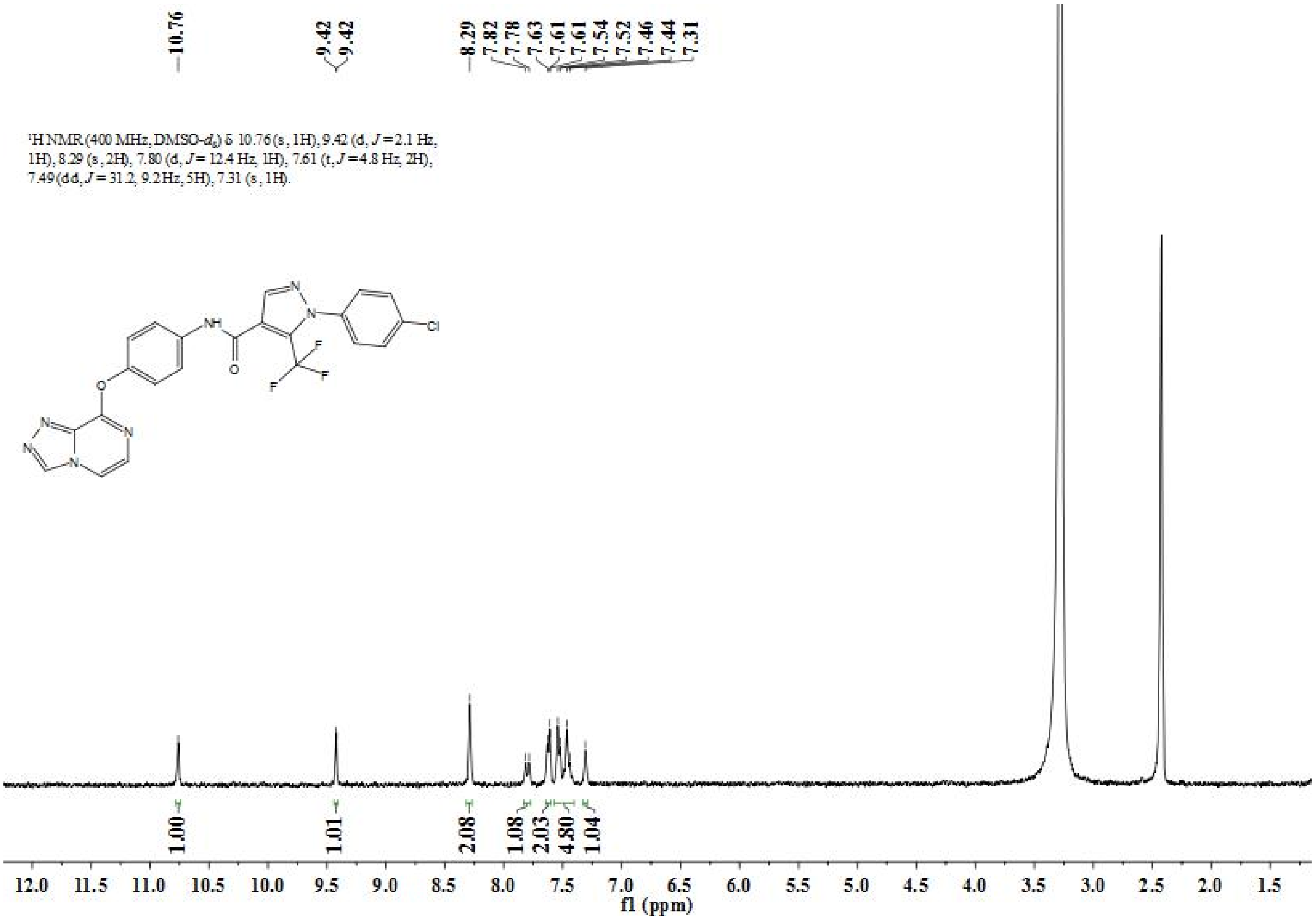

Figure 4. ^{1}H NMR spectra of compound **16d.**

N-(4-([1,2,4]triazolo[4,3-*a*]pyrazin-8-yloxy)phenyl)-3-(thiophen-2-yl)-1H-pyrazole-5-carboxamide (16e)

Yellow solid in 22.6%, M.P.: 251.6–253.7 °C. ^{1}H NMR (400 MHz, DMSO-d_6) δ 13.88 (s, 1H), 9.47 (s, 1H), 8.31 (d, J = 4.9 Hz, 2H), 7.89 (d, J = 20.5 Hz, 2H), 7.68 (d, J = 8.5 Hz, 1H), 7.37 (s, 4H), 7.31 (s, 1H), 7.16 (d, J = 17.3 Hz, 1H). TOF MS ES+ (m/z): [M + H]$^+$, calcd for $C_{19}H_{13}N_7O_2S$: 404.0930, found, 404.0851.

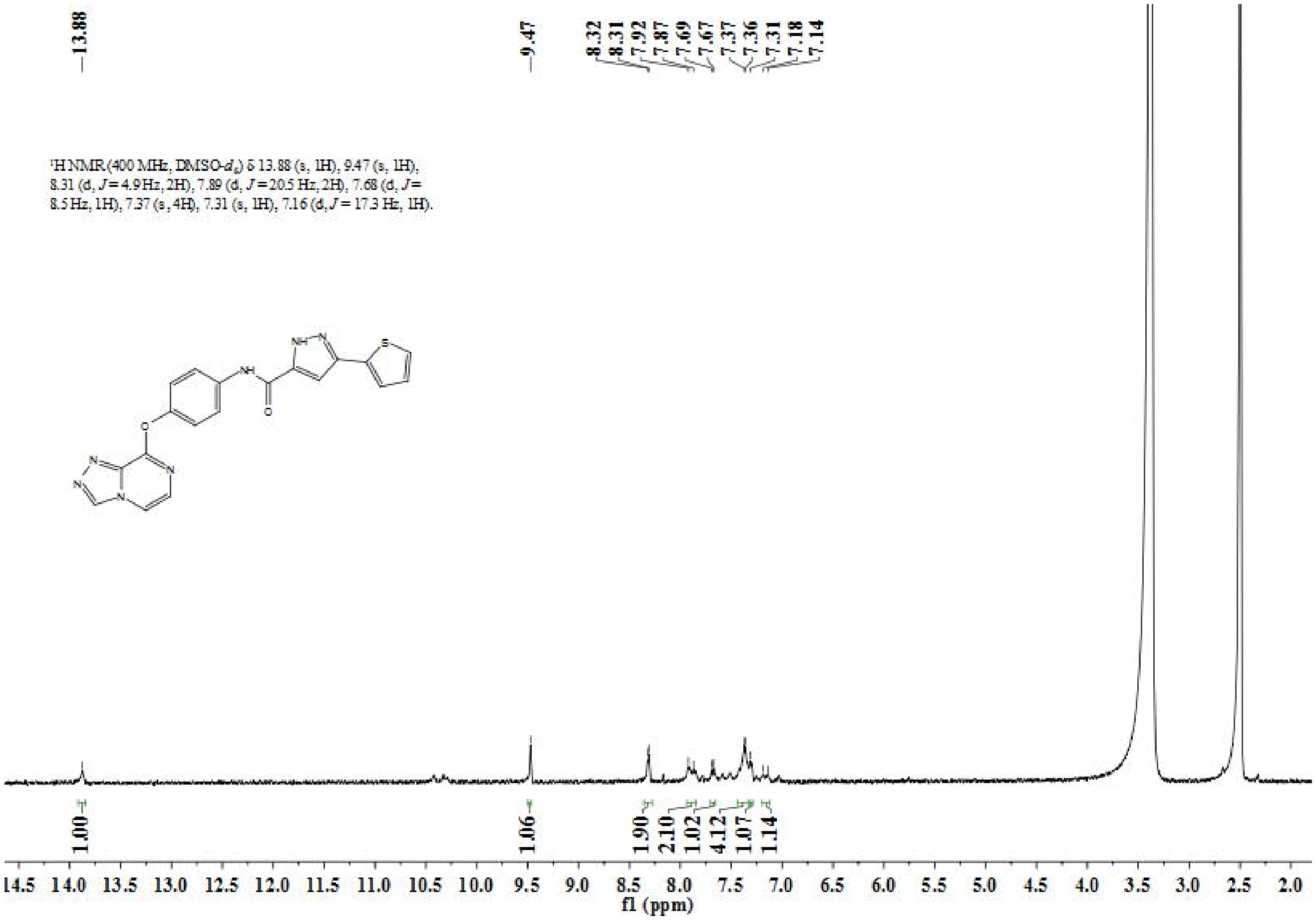

Figure 5. ^{1}H NMR spectra of compound **16e.**

N-(4-([1,2,4]triazolo[4,3-*a*]pyrazin-8-yloxy)phenyl)-3-(thiophen-2-yl)isoxazole-5-carboxamide (16f)

Yellow solid in 25.1%, M.P.: 291.5–293.1 °C. ^{1}H NMR (400 MHz, DMSO-d_6) δ 11.11 (s, 1H), 9.51 (s, 1H), 8.37 (d, *J* = 4.7 Hz, 1H), 8.04–7.83 (m, 4H), 7.72 (d, *J* = 9.0 Hz, 1H), 7.57–7.50 (m, 1H), 7.39 (d, *J* = 6.2 Hz, 2H), 7.30 (d, *J* = 4.0 Hz, 1H). TOF MS ES+ (*m/z*): [M + H]$^{+}$, calcd for $C_{19}H_{12}N_6O_3S$: 405.0692, found, 405.0723.

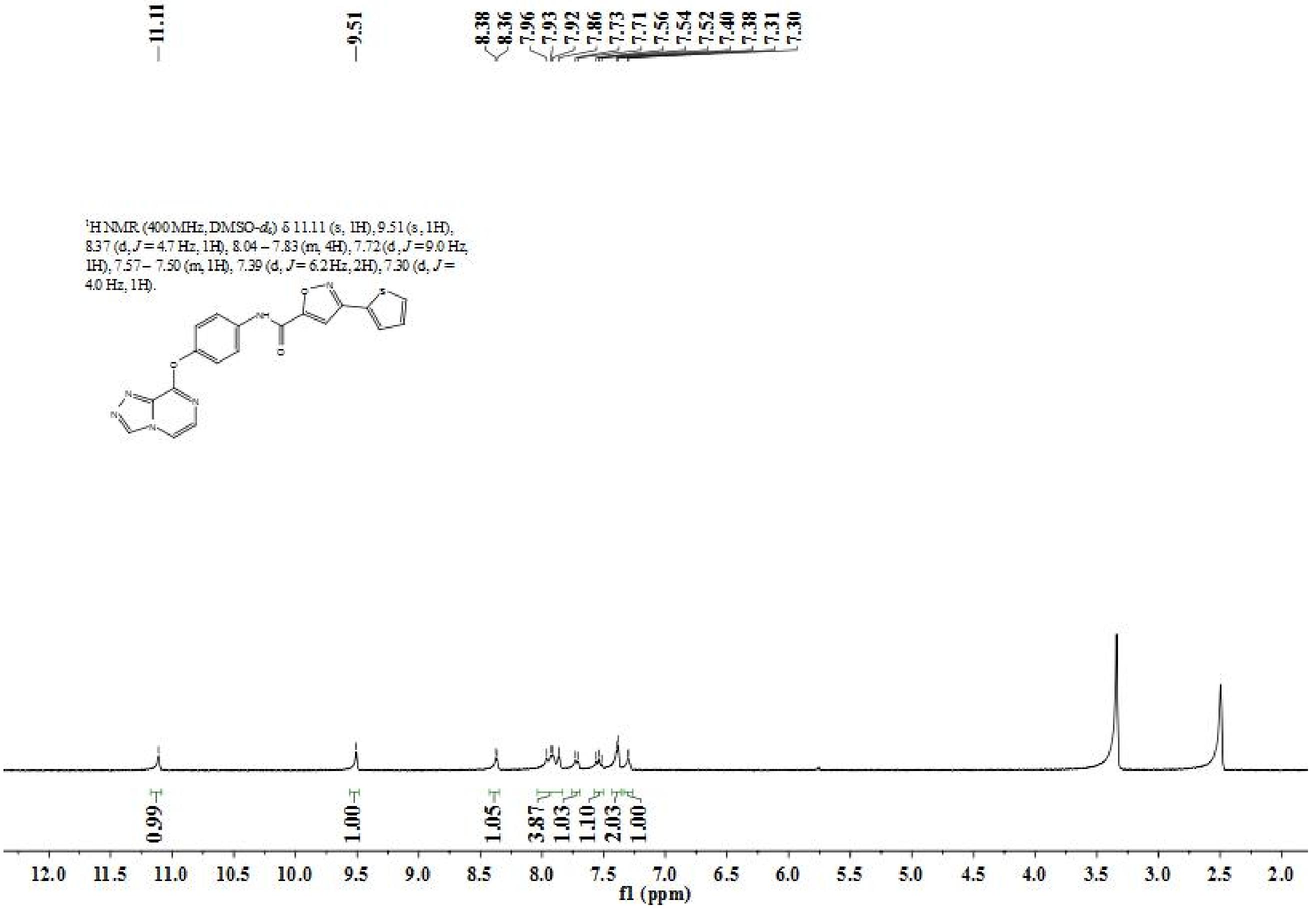

Figure 6. ^{1}H NMR spectra of compound **16f.**

N-(4-([1,2,4]triazolo[4,3-*a*]pyrazin-8-yloxy)-3-fluorophenyl)-4-methyl-2-phenylthiazole-5-carboxamide (16g)

Yellow solid in 48.3%, M.P.: 278.1–282.7 °C. ^{1}H NMR (400 MHz, DMSO-d_6) δ 10.43 (s, 1H), 9.48 (s, 1H), 8.33 (s, 1H), 8.00 (s, 2H), 7.80 (s, 2H), 7.55 (s, 3H), 7.36 (s, 2H), 2.68 (s, 3H). TOF MS ES+ (*m/z*): [M + H]$^+$, calcd for $C_{22}H_{15}FN_6O_2S$: 447.1039, found, 447.0961.

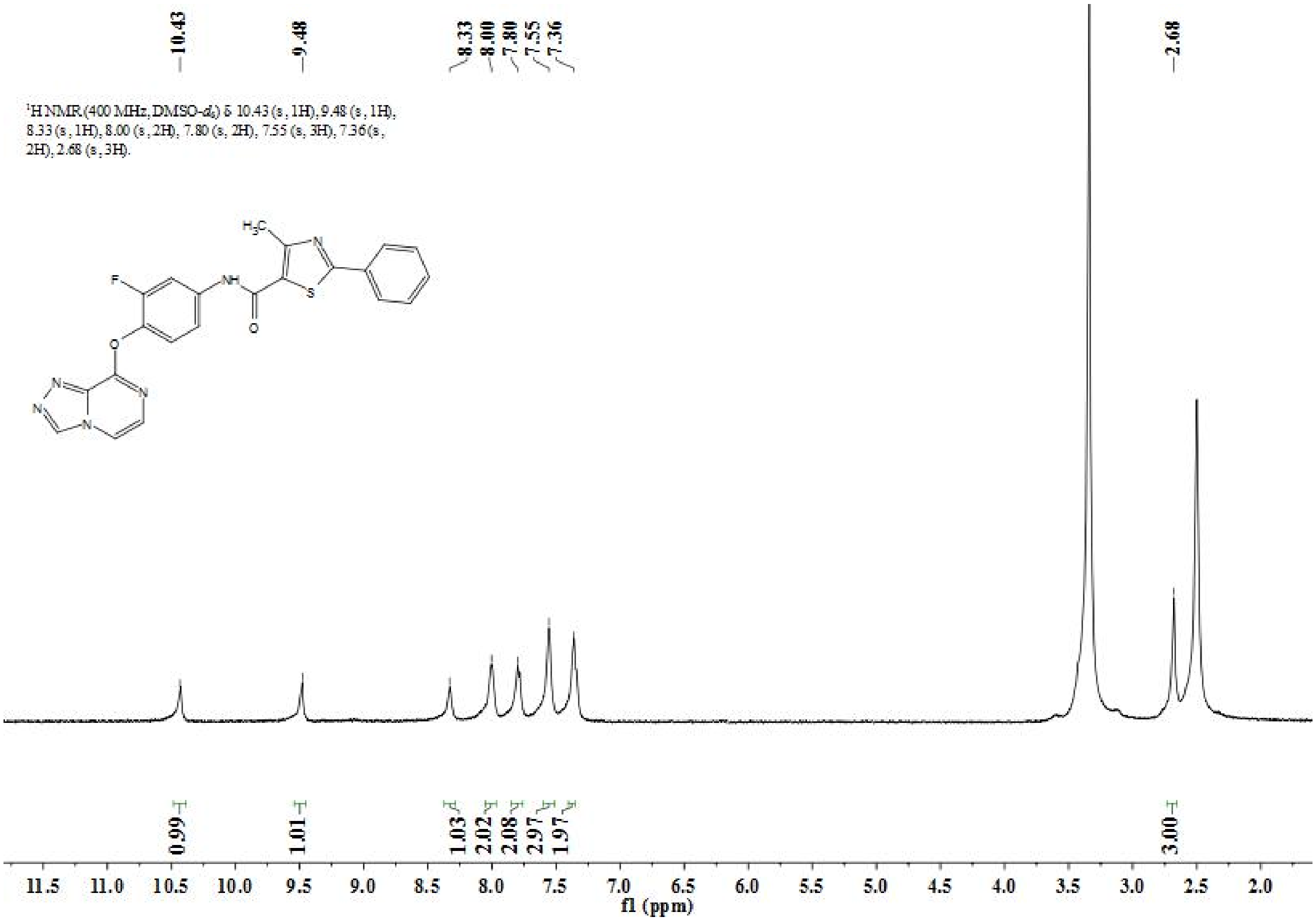

Figure 7. ^{1}H NMR spectra of compound **16g.**

N-(4-([1,2,4]triazolo[4,3-*a*]pyrazin-8-yloxy)-3-fluorophenyl)-4-methyl-2-(pyridin-2-yl)thiazole-5-carboxamide (16h)

Yellow solid in 57.3%, M.P.: 203.4–207.6 °C. ^{1}H NMR (400 MHz, DMSO-d_6) δ 10.66 (s, 1H), 9.51 (s, 1H), 8.68 (d, J = 5.0 Hz, 1H), 8.37 (d, *J* = 5.1 Hz, 1H), 8.18 (d, *J* = 8.0 Hz, 1H), 8.03 (t, *J* = 7.4 Hz, 1H), 7.88 (d, *J* = 13.4 Hz, 1H), 7.63–7.51 (m, 3H), 7.39 (d, *J* = 4.8 Hz, 2H), 2.69 (s, 3H). TOF MS ES+ (*m/z*): $[M + H]^+$, calcd for $C_{21}H_{14}FN_7O_2S$: 448.0992, found, 448.0914.

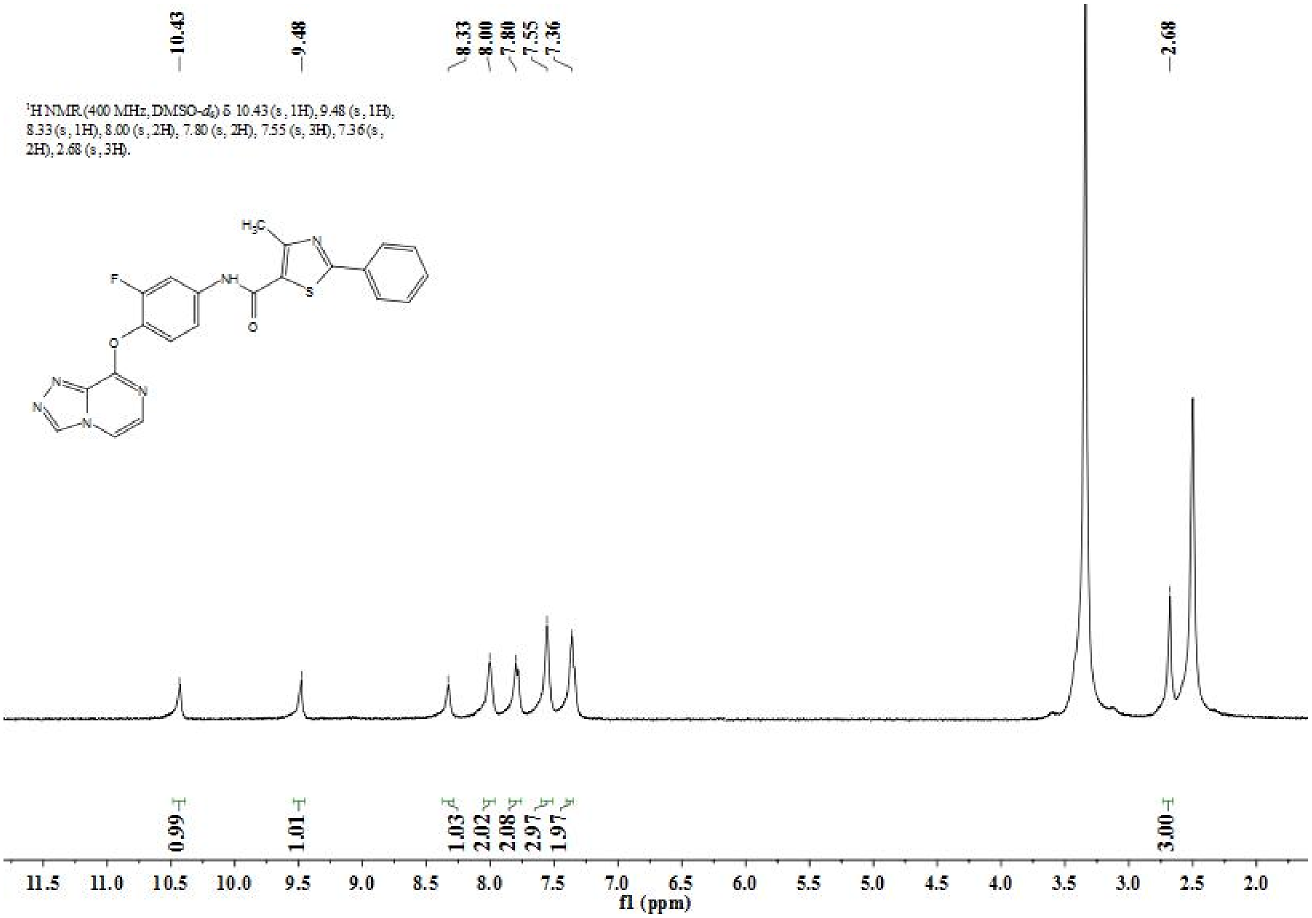

Figure 8. ^{1}H NMR spectra of compound **16h.**

N-(4-([1,2,4]triazolo[4,3-*a*]pyrazin-8-yloxy)-3-fluorophenyl)-4-methyl-2-(pyridin-3-yl)thiazole-5-carboxamide (16i)

Yellow solid in 57.6%, M.P.: 224.3–226.8 °C. ^{1}H NMR (400 MHz, DMSO-d_6) δ 10.65 (s, 1H), 9.51 (s, 1H), 9.19 (s, 1H), 8.73 (s, 1H), 8.37 (d, J = 5.5 Hz, 2H), 7.87 (d, J = 13.6 Hz, 2H), 7.57 (d, J = 8.2 Hz, 2H), 7.40 (s, 1H), 2.70 (s, 3H). TOF MS ES+ (m/z): $[M + H]^+$, calcd for $C_{21}H_{14}FN_7O_2S$: 448.0992, found, 448.0914.

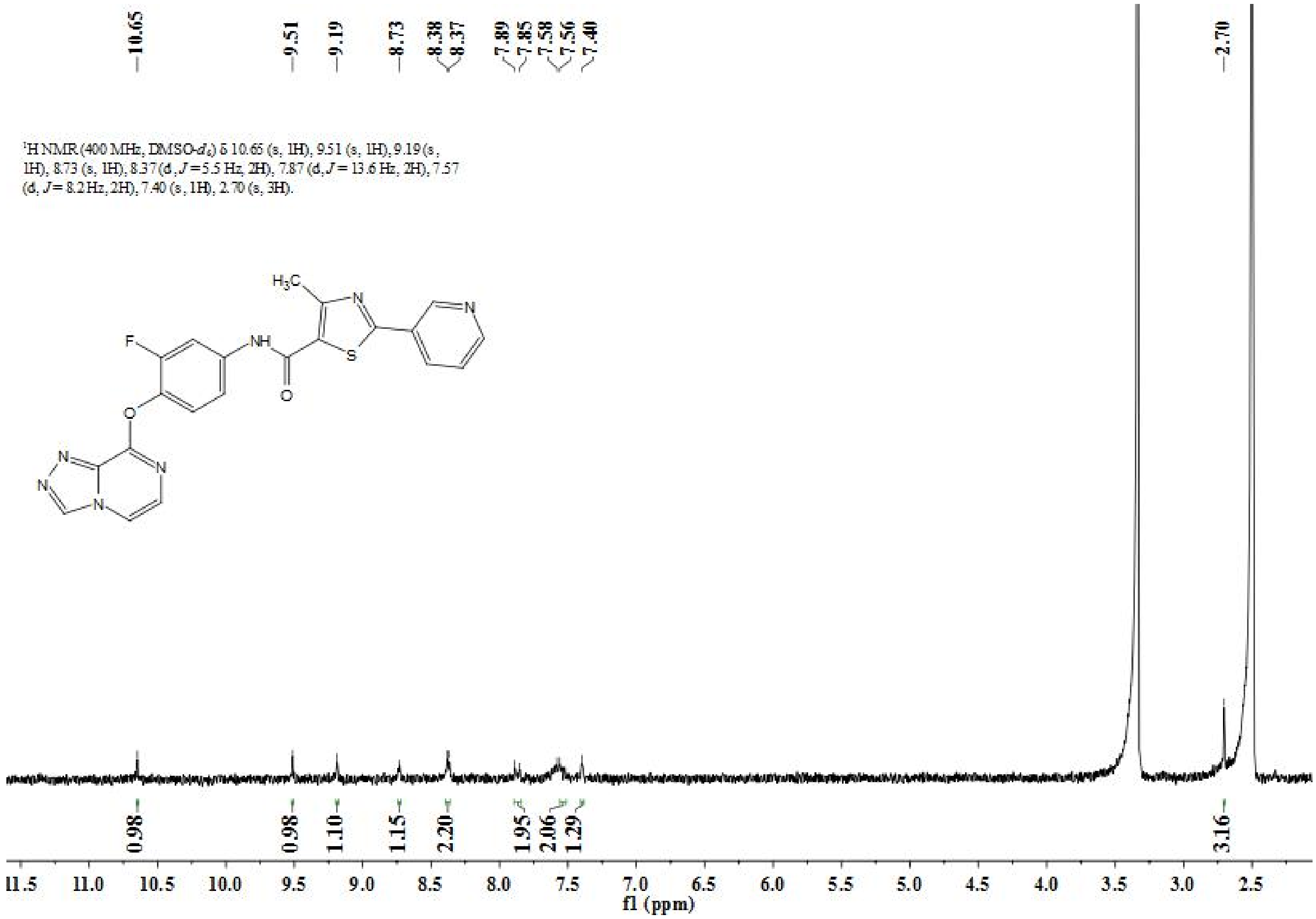

Figure 9. ^{1}H NMR spectra of compound **16i.**

N-(4-([1,2,4]triazolo[4,3-*a*]pyrazin-8-yloxy)-3-fluorophenyl)-4-methyl-2-(pyridin-4-yl)thiazole-5-carboxamide (16j)

Yellow solid in 27.4%, M.P.: 226.9–229.1 °C. ^{1}H NMR (400 MHz, DMSO-d_6) δ 10.70 (s, 1H), 9.51 (s, 1H), 8.76 (d, *J* = 5.5 Hz, 2H), 8.37 (d, *J* = 4.8 Hz, 1H), 7.94 (d, *J* = 5.9 Hz, 2H), 7.87 (d, *J* = 12.4 Hz, 1H), 7.54 (d, *J* = 11.9 Hz, 2H), 7.39 (d, *J* = 4.8 Hz, 1H), 2.70 (s, 3H). TOF MS ES+ (*m/z*): [M + H]$^+$, calcd for $C_{21}H_{14}FN_7O_2S$: 448.0992, found, 448.0914.

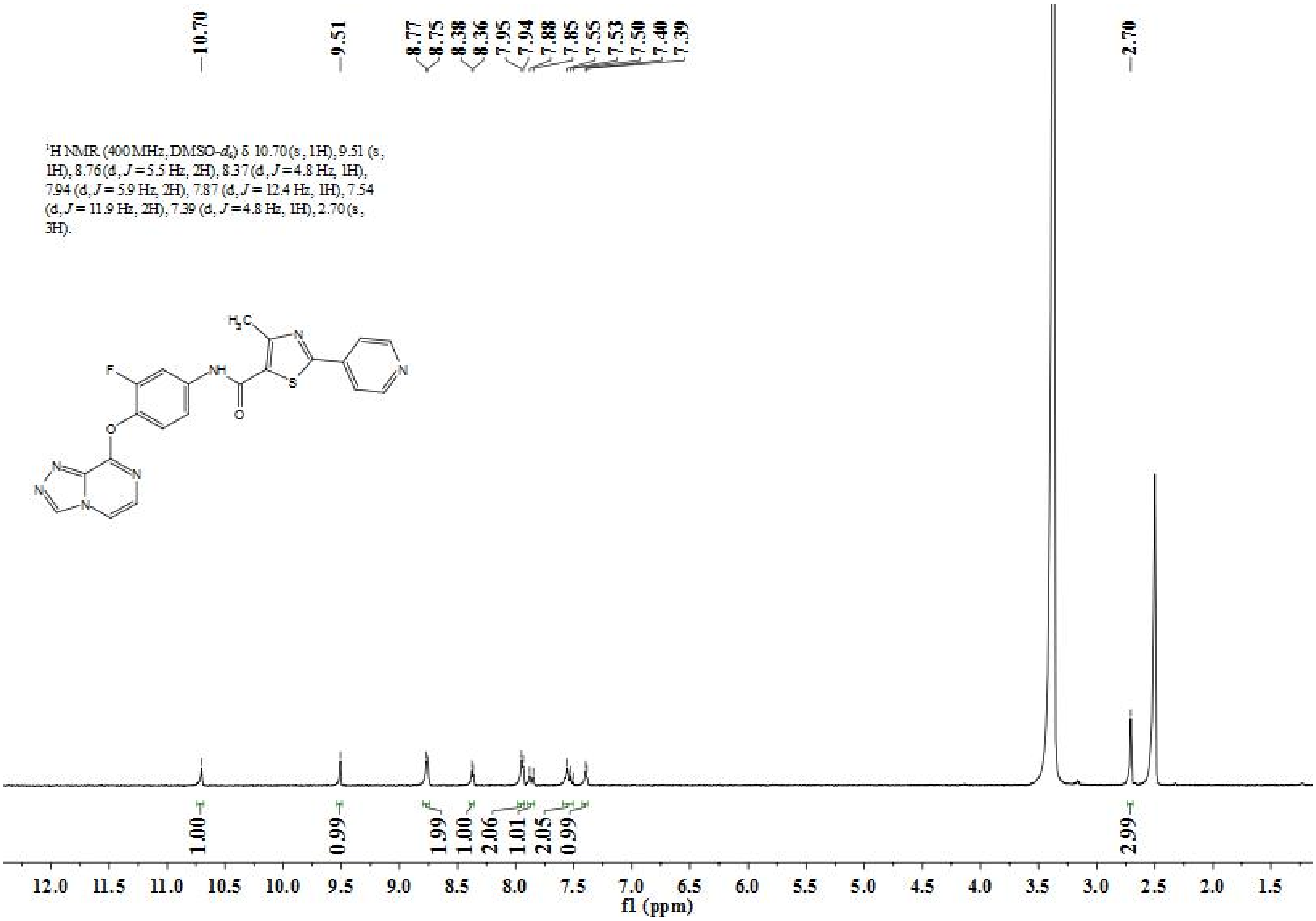

Figure 10. ^{1}H NMR spectra of compound **16j.**

N-(4-([1,2,4]triazolo[4,3-*a*]pyrazin-8-yloxy)-3-fluorophenyl)-1-(4-chlorophenyl)-5-(trifluoromethyl)-1H-pyrazole-4-carboxamide (16k)

Yellow solid in 38.5%, M.P.: 252.1–253.7 °C. ^{1}H NMR (400 MHz, DMSO-d_6) δ 10.87 (s, 1H), 9.51 (s, 1H), 8.38 (d, *J* = 7.8 Hz, 2H), 7.89 (d, *J* = 12.6 Hz, 1H), 7.70 (d, *J* = 8.4 Hz, 2H), 7.62 (d, *J* = 8.4 Hz, 2H), 7.54 (d, *J* = 9.6 Hz, 2H), 7.39 (d, *J* = 4.8 Hz, 1H). TOF MS ES+ (*m/z*): [M + H]$^+$, calcd for $C_{22}H_{12}ClF_4N_7O_2$: 518.0755, found, 518.0677.

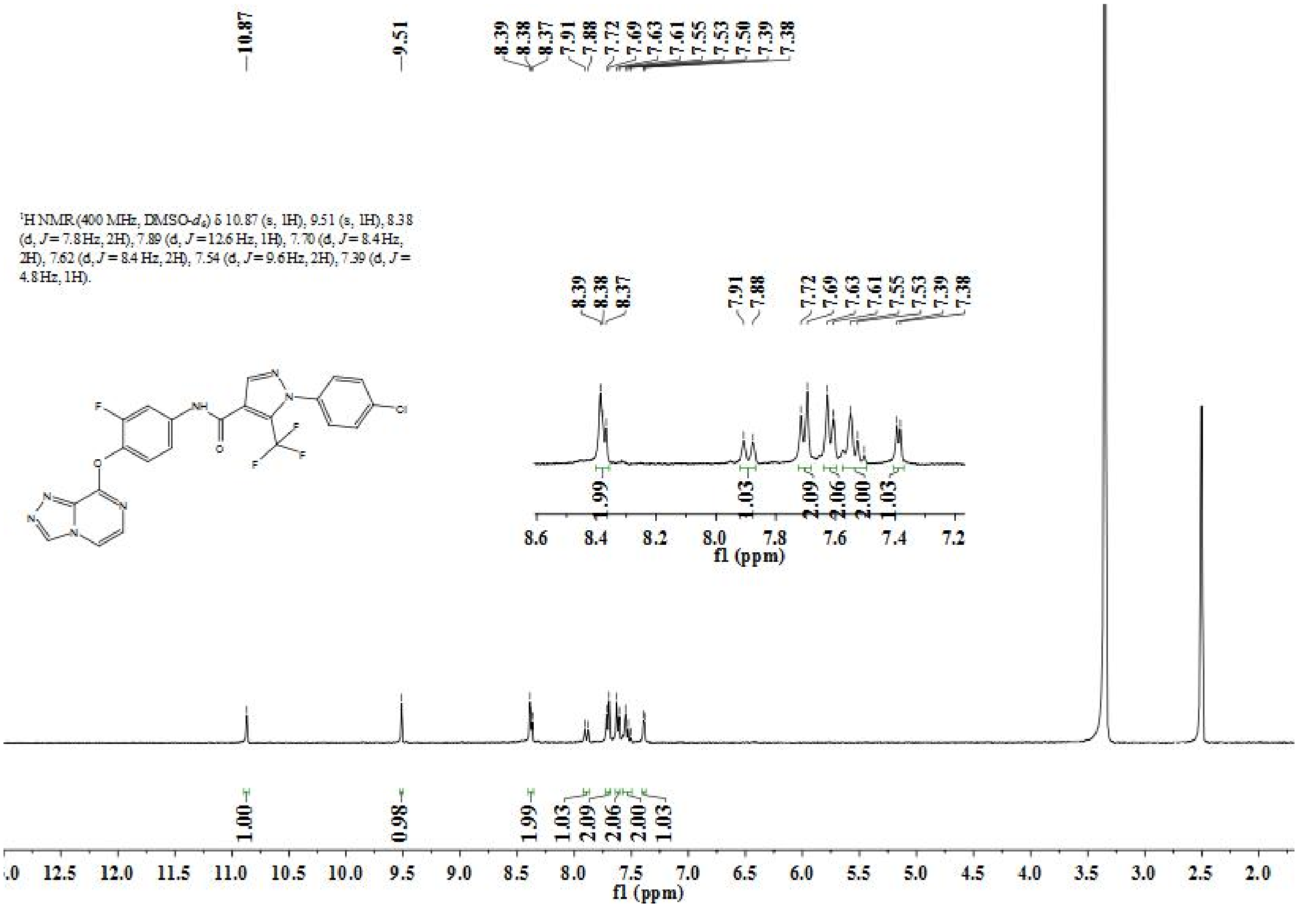

Figure 11. ^{1}H NMR spectra of compound **16k.**

N-(4-([1,2,4]triazolo[4,3-*a*]pyrazin-8-yloxy)-3-fluorophenyl)-3-(thiophen-2-yl)isoxazole-5-carboxamide (16l)

Yellow solid in 41.3%, M.P.: 293.3–295.1 °C. ^{1}H NMR (400 MHz, DMSO-d_6) δ 10.94 (s, 1H), 9.49 (s, 1H), 8.33 (s, 1H), 7.90 (d, *J* = 9.5 Hz, 4H), 7.38 (d, *J* = 6.1 Hz, 3H), 7.31 (s, 1H). TOF MS ES+ (*m/z*): [M + H]$^+$, calcd for $C_{19}H_{11}FN_6O_3S$: 423.0676, found, 423.0597.

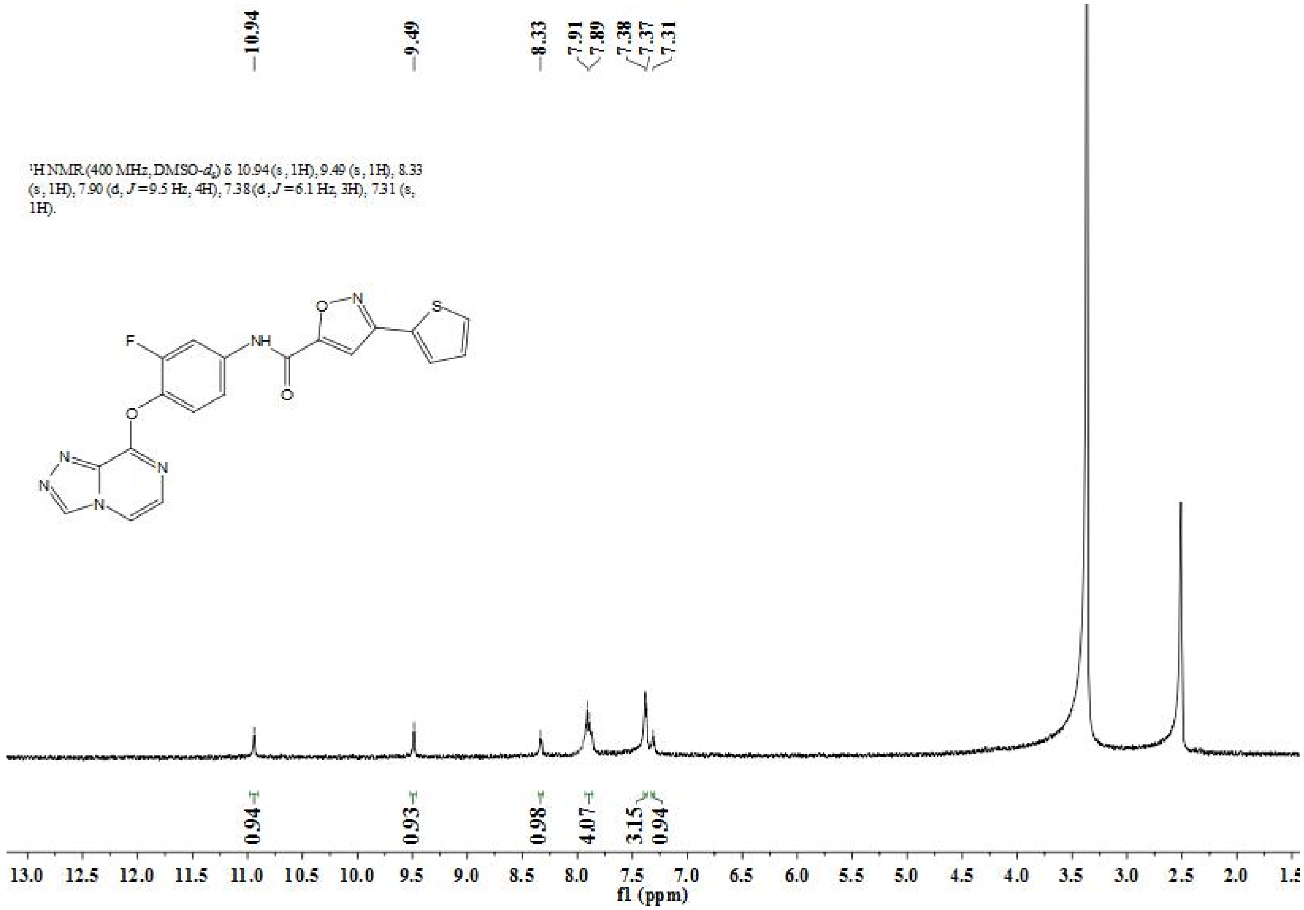

Figure 12. ^{1}H NMR spectra of compound **16l.**

4-methyl-N-(4-((3-methyl-[1,2,4]triazolo[4,3-*a*]pyrazin-8-yl)oxy)phenyl)-2-phenylthiazole-5-carboxamide (17a)

Light yellow solid in in 47.9%, M.P.: 293.5–296.1 °C. ^{1}H NMR (400 MHz, DMSO-d_6) δ 10.32 (s, 1H), 8.08 (d, J = 4.8 Hz, 1H), 7.88 (d, *J* = 3.9 Hz, 2H), 7.67 (d, *J* = 9.4 Hz, 2H), 7.44 (d, *J* = 3.2 Hz, 3H), 7.23 (dd, *J* = 13.5, 6.9 Hz, 3H), 2.62 (s, 3H), 2.56 (s, 3H). TOF MS ES+ (*m/z*): [M + H]$^+$, calcd for $C_{23}H_{18}N_6O_2S$: 443.1290, found, 443.1312.

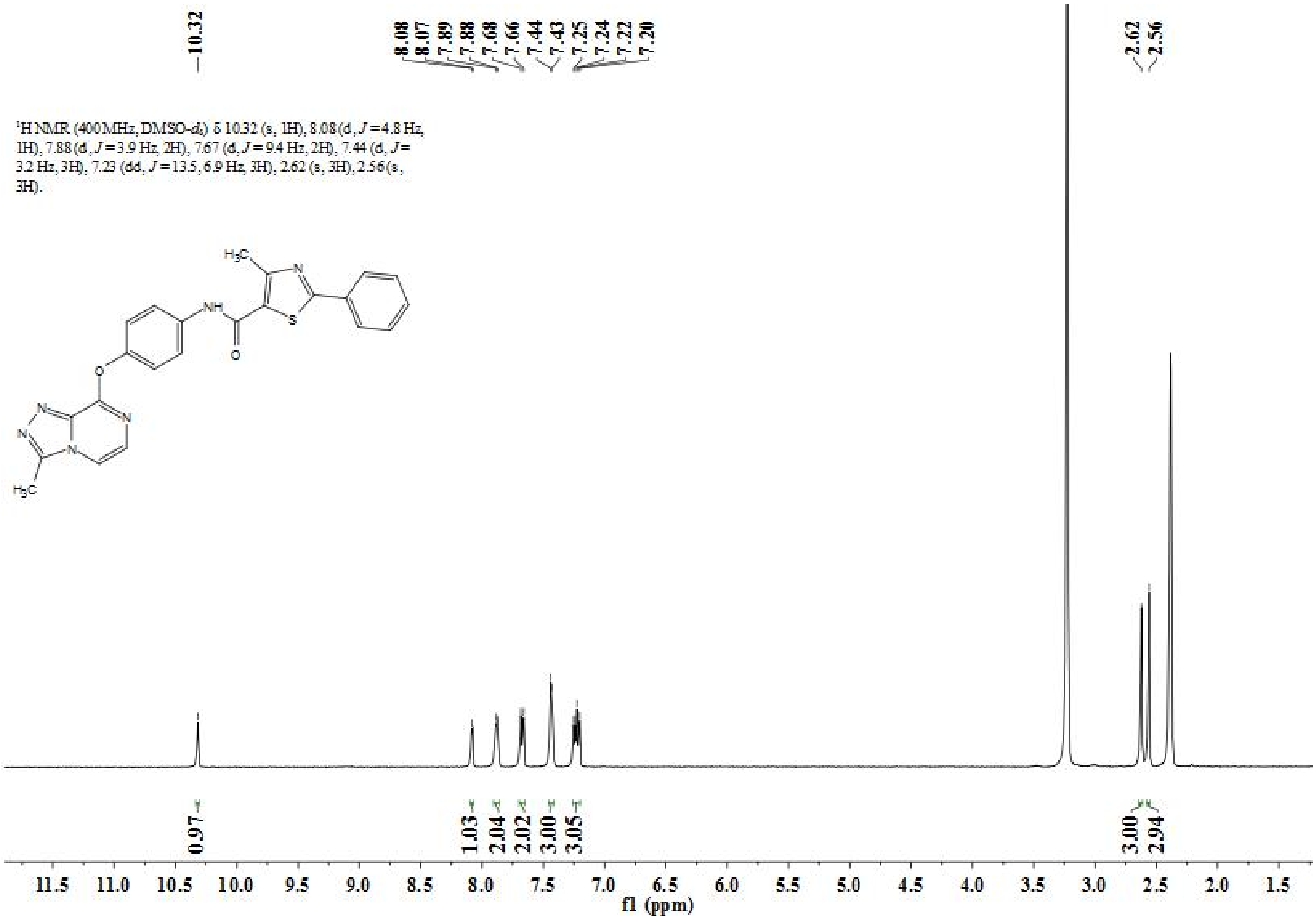

Figure 13. ^{1}H NMR spectra of compound **17a.**

4-methyl-N-(4-((3-methyl-[1,2,4]triazolo[4,3-*a*]pyrazin-8-yl)oxy)phenyl)-2-(pyridin-2-yl)thiazole-5-carboxamide (17b)

Yellow solid in 20.8%, M.P.: 215.4–217.5 °C. ^{1}H NMR (400 MHz, DMSO-d_6) δ 10.44 (s, 1H), 8.68 (s, 1H), 8.18 (d, *J* = 9.9 Hz, 2H), 8.02 (d, *J* = 7.8 Hz, 1H), 7.79 (d, *J* = 8.5 Hz, 2H), 7.57 (s, 1H), 7.38–7.31 (m, 3H), 2.74 (s, 3H), 2.69 (s, 3H). TOF MS ES+ (*m/z*): [M + H]$^+$, calcd for $C_{21}H_{17}N_7O_2S$: 444.1243, found, 444.1164.

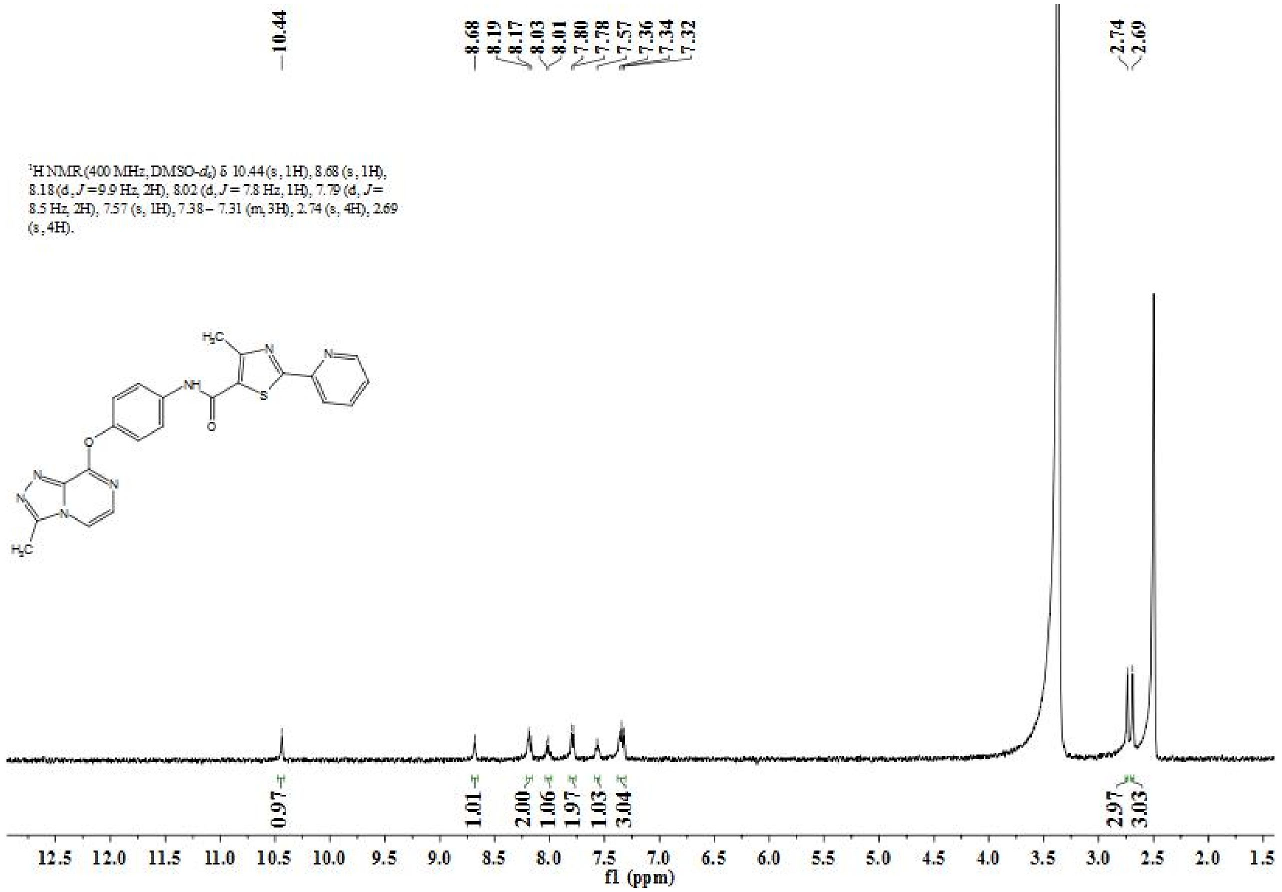

Figure 14. ^{1}H NMR spectra of compound **17b.**

4-methyl-N-(4-((3-methyl-[1,2,4]triazolo[4,3-*a*]pyrazin-8-yl)oxy)phenyl)-2-(pyridin-4-yl)thiazole-5-carboxamide (17c)

Yellow solid in 43.1%, M.P.: 270.7–273.8 °C. ^{1}H NMR (400 MHz, DMSO-d_6) δ 10.58 (s, 1H), 8.73 (d, J = 5.0 Hz, 2H), 8.62 (d, J = 5.1 Hz, 1H), 8.18 (d, J = 4.9 Hz, 1H), 7.91 (d, J = 5.0 Hz, 2H), 7.77 (d, J = 8.0 Hz, 2H), 7.36–7.32 (m, 2H), 2.72 (s, 3H), 2.68 (s, 3H). TOF MS ES+ (m/z): $[M + H]^+$, calcd for $C_{22}H_{17}N_7O_2S$: 444.1243, found, 444.1356.

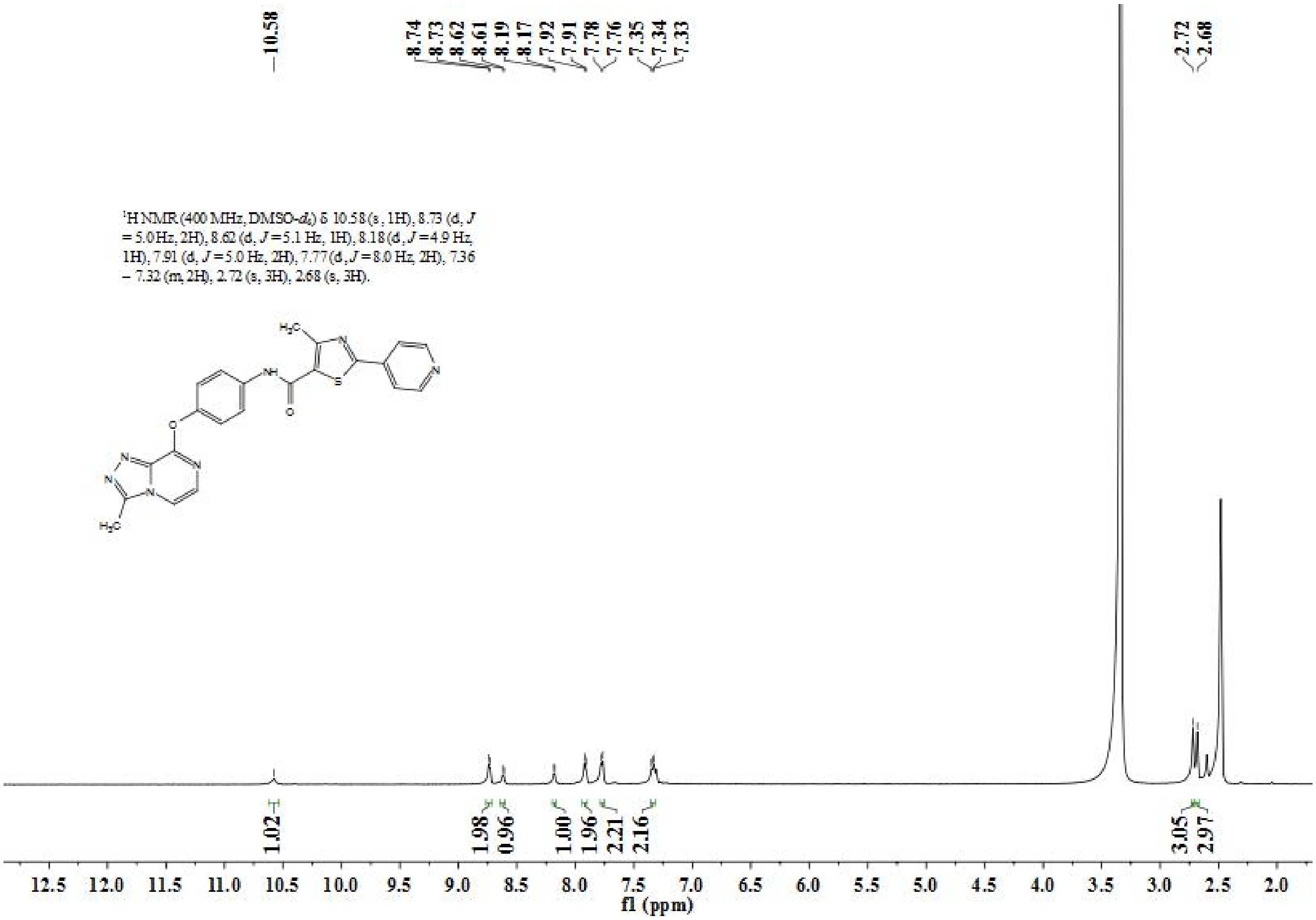

Figure 15. ^{1}H NMR spectra of compound **17c.**

2-(4-fluorophenyl)-4-methyl-N-(4-((3-methyl-[1,2,4]triazolo[4,3-*a*]pyrazin-8-yl)oxy)phenyl)thiazole-5-carboxamide (17d)

Yellow solid in 43.6%, M.P.: 300.4–302.0 °C. ^{1}H NMR (400 MHz, DMSO-d_6) δ 10.97 (s, 1H), 8.19 (s, 1H), 8.05 (s, 2H), 7.89–7.74 (m, 2H), 7.47–7.26 (m, 5H), 2.74 (s, 3H), 2.67 (s, 3H). TOF MS ES+ (*m/z*): $[M + H]^+$, calcd for $C_{23}H_{17}FN_6O_2S$: 461.1196, found, 461.1126.

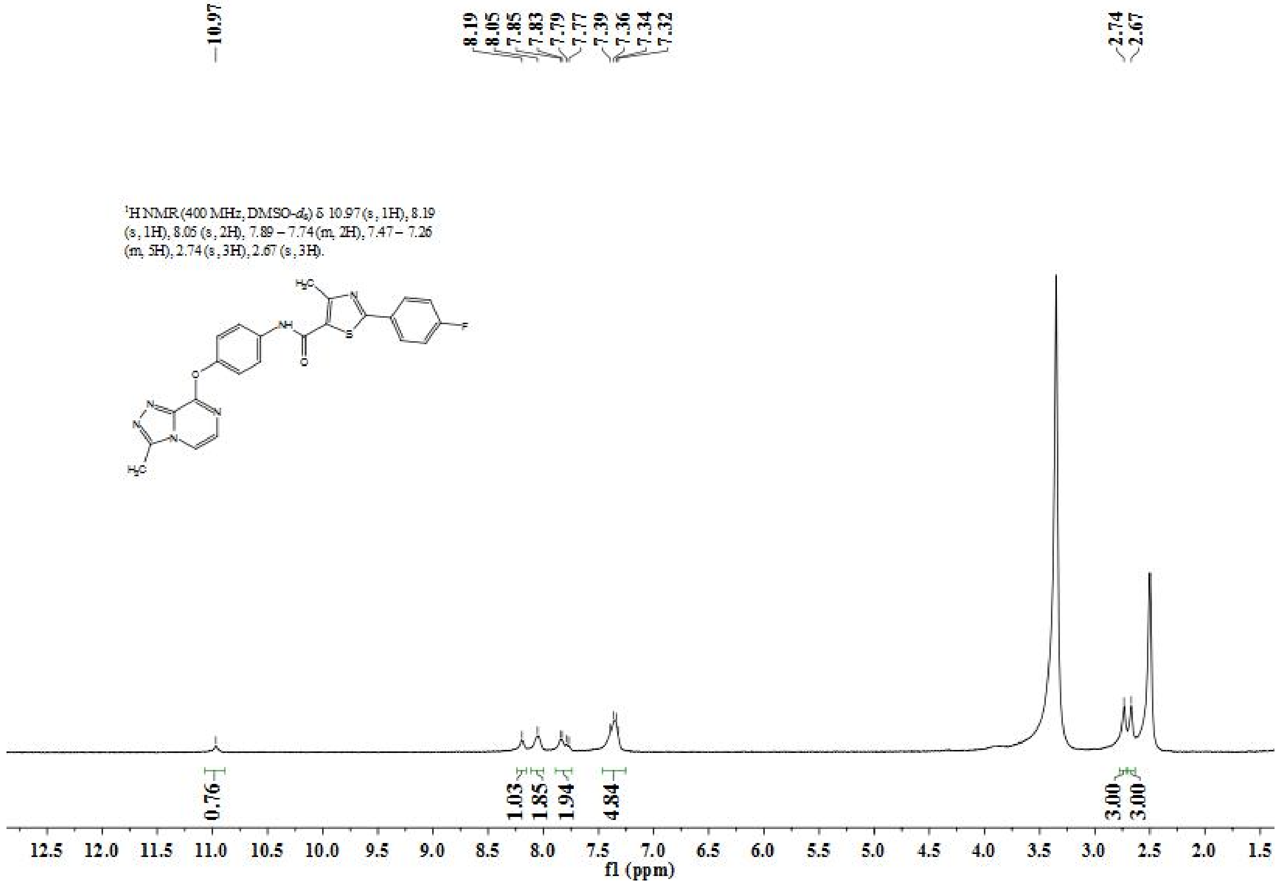

Figure 16. ^{1}H NMR spectra of compound **17d.**

1-(4-chlorophenyl)-N-(4-((3-methyl-[1,2,4]triazolo[4,3-*a*]pyrazin-8-yl)oxy)phenyl)-5-(trifluoromethyl)-1H-pyrazole-4-carboxamide (17e)

Yellow solid in 75.2%, M.P.: 288.9–289.5 °C. ^{1}H NMR (400 MHz, DMSO-d_6) δ 10.72 (s, 1H), 8.39 (s, 1H), 8.19 (d, *J* = 4.8 Hz, 1H), 7.81 (d, *J* = 8.5 Hz, 2H), 7.70 (d, *J* = 8.6 Hz, 2H), 7.61 (d, *J* = 8.2 Hz, 2H), 7.39–7.30 (m, 3H), 2.74 (s, 3H). TOF MS ES+ (*m/z*): $[M + H]^+$, calcd for $C_{23}H_{15}ClF_3N_7O_2$: 514.1006, found, 513.9928.

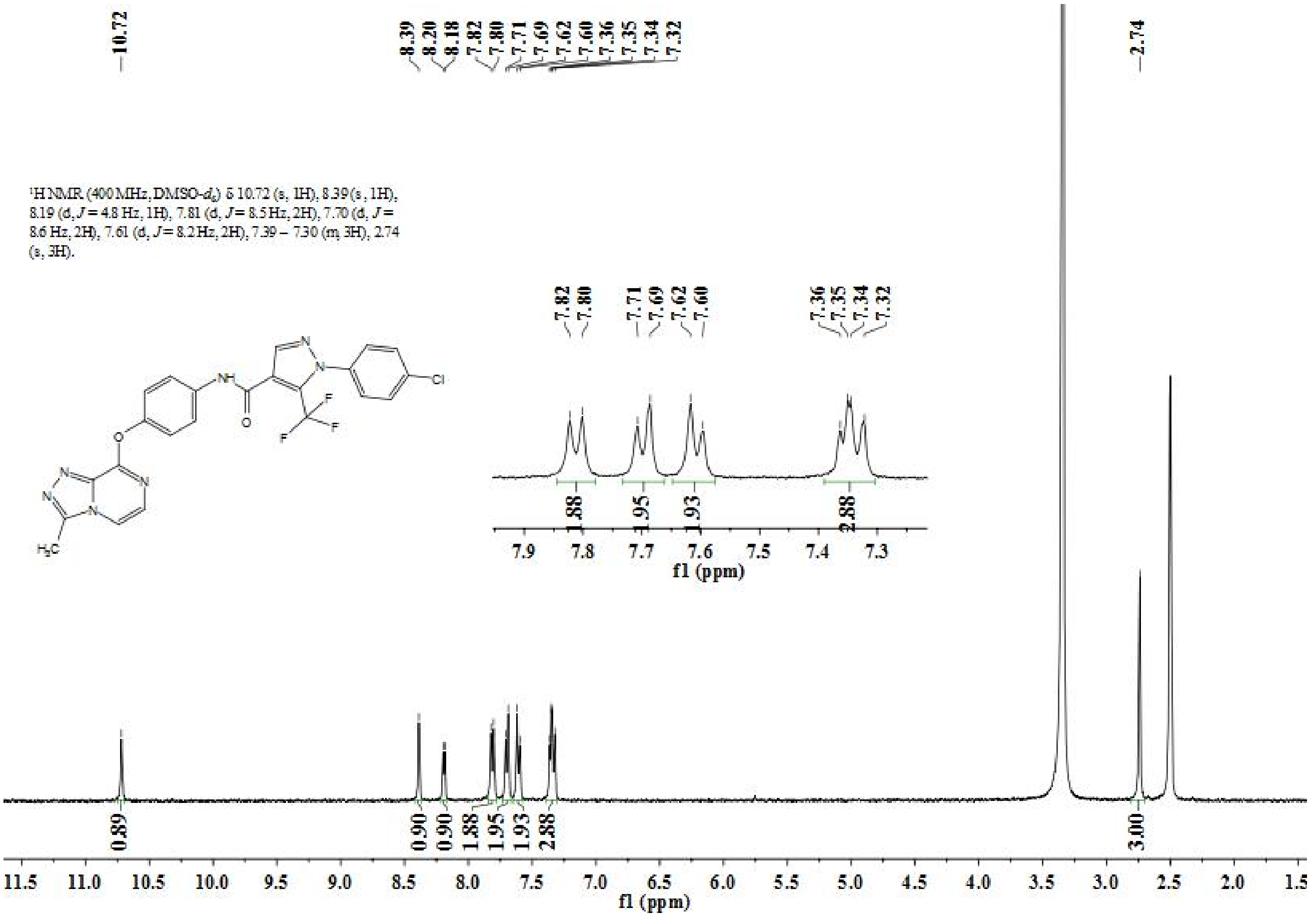

Figure 17. ^{1}H NMR spectra of compound **17e.**

N-(3-fluoro-4-((3-methyl-[1,2,4]triazolo[4,3-*a*]pyrazin-8-yl)oxy)phenyl)-3-(thiophen-2-yl)-1H-pyrazole-5-carboxamide (17f)

Light yellow solid in 29.3%, M.P.: 275.6–278.1 °C. ^{1}H NMR (400 MHz, DMSO-d_6) δ 13.86 (s, 1H), 10.40 (s, 1H), 8.20–8.14 (m, 1H), 7.93–7.80 (m, 2H), 7.67–7.46 (m, 2H), 7.42–7.30 (m, 3H), 7.31–7.21 (m, 1H), 7.15 (dd, *J* = 22.1, 4.6 Hz, 1H), 2.72 (s, 3H). TOF MS ES+ (*m/z*): [M + H]$^+$, calcd for $C_{20}H_{15}N_7O_2S$: 418.1086, found, 418.1008.

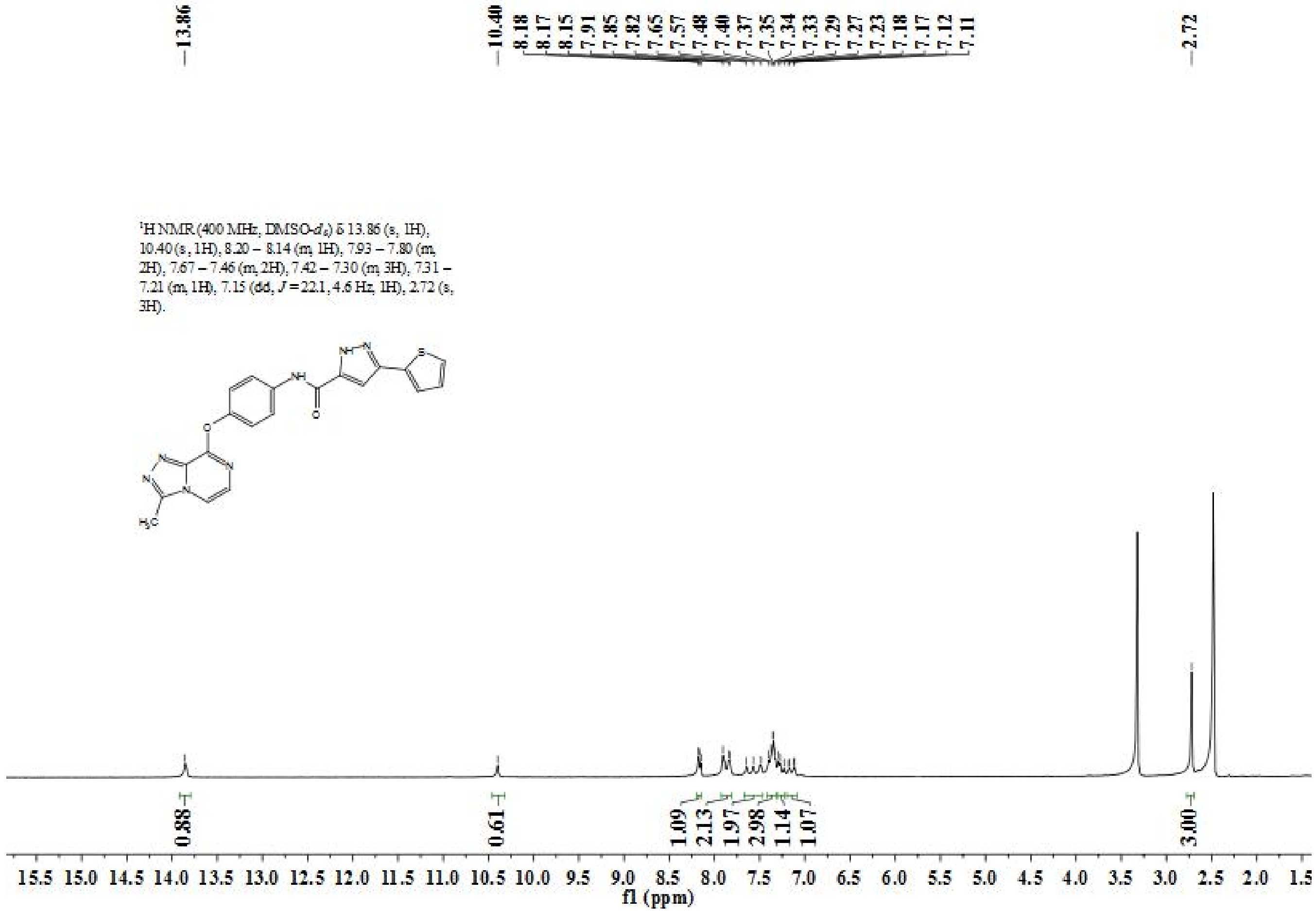

Figure 18. ^{1}H NMR spectra of compound **17f.**

N-(3-fluoro-4-((3-methyl-[1,2,4]triazolo[4,3-*a*]pyrazin-8-yl)oxy)phenyl)-4-methyl-2-phenylthiazole-5-carboxamide (17g)

Yellow solid in 46.2%, M.P.: 299.0–302.1 °C. ^{1}H NMR (400 MHz, DMSO-d_6) δ 10.57 (s, 1H), 8.24 (d, J = 4.8 Hz, 1H), 8.01 (d, J = 4.1 Hz, 2H), 7.86 (d, J = 12.8 Hz, 1H), 7.56 (d, J = 2.6 Hz, 4H), 7.51 (t, J = 8.6 Hz, 1H), 7.39 (d, J = 4.8 Hz, 1H), 2.75 (s, 3H), 2.69 (s, 3H). TOF MS ES+ (*m/z*): $[M + H]^+$, calcd for $C_{23}H_{17}FN_6O_2S$: 461.1196, found, 460.9986.

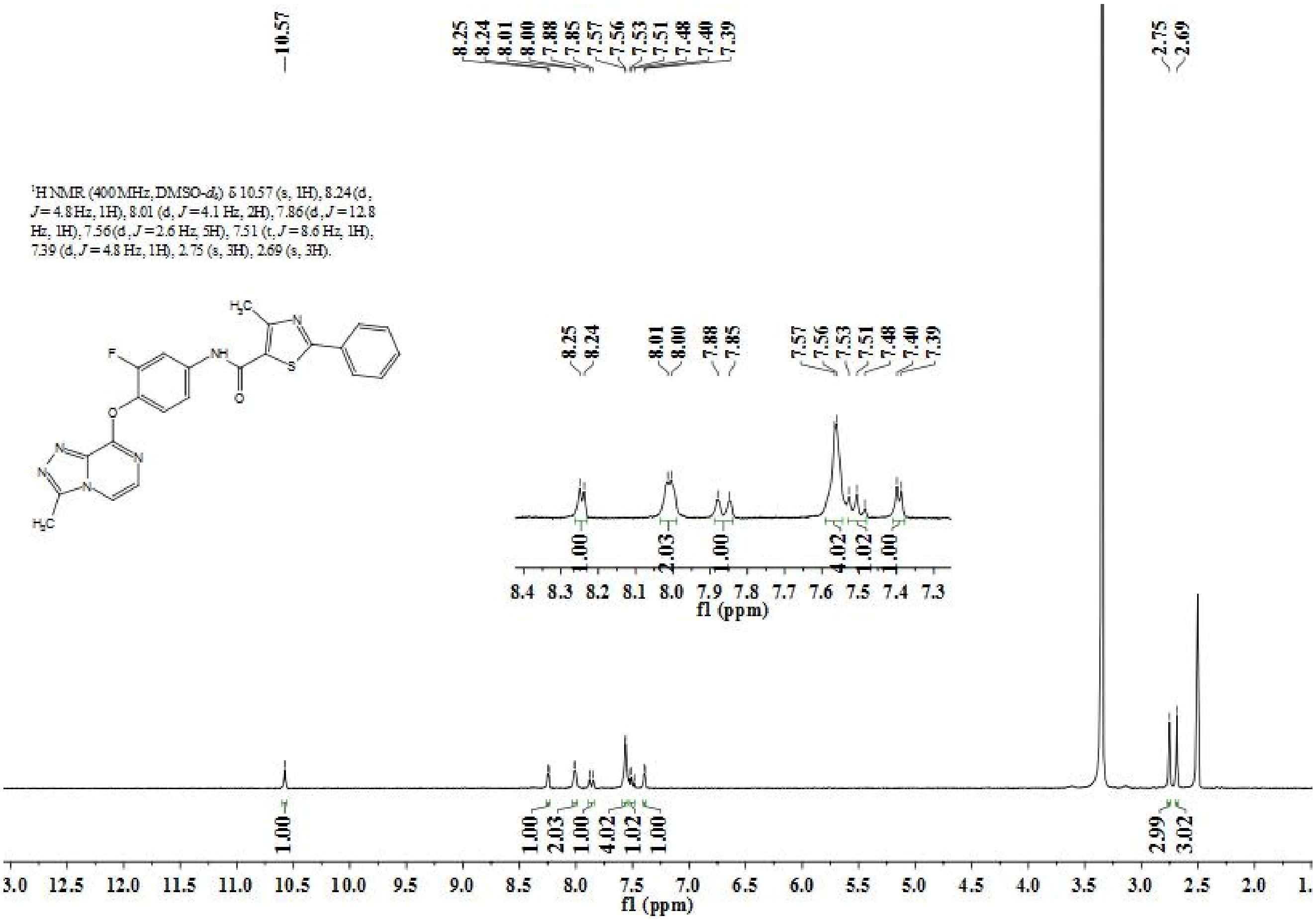

Figure 19. ^{1}H NMR spectra of compound **17g.**

N-(3-fluoro-4-((3-methyl-[1,2,4]triazolo[4,3-*a*]pyrazin-8-yl)oxy)phenyl)-4-methyl-2-(pyridin-2-yl)thiazole-5-carboxamide (17h)

Yellow solid in 29.6%, M.P.: 195.8–198.3 °C. ^{1}H NMR (400 MHz, DMSO-d_6) δ 10.60 (s, 1H), 8.69 (s, 1H), 8.24 (d, J = 4.8 Hz, 1H), 8.18 (d, J = 7.9 Hz, 1H), 8.02 (d, J = 7.6 Hz, 1H), 7.87 (d, J = 12.1 Hz, 1H), 7.58 (d, J = 7.2 Hz, 2H), 7.51 (d, J = 8.5 Hz, 1H), 7.39 (d, J = 4.6 Hz, 1H), 2.75 (s, 3H), 2.69 (s, 3H). TOF MS ES+ (*m/z*): [M + H]$^+$, calcd for $C_{22}H_{16}FN_7O_2S$: 462.1148, found, 462.1070.

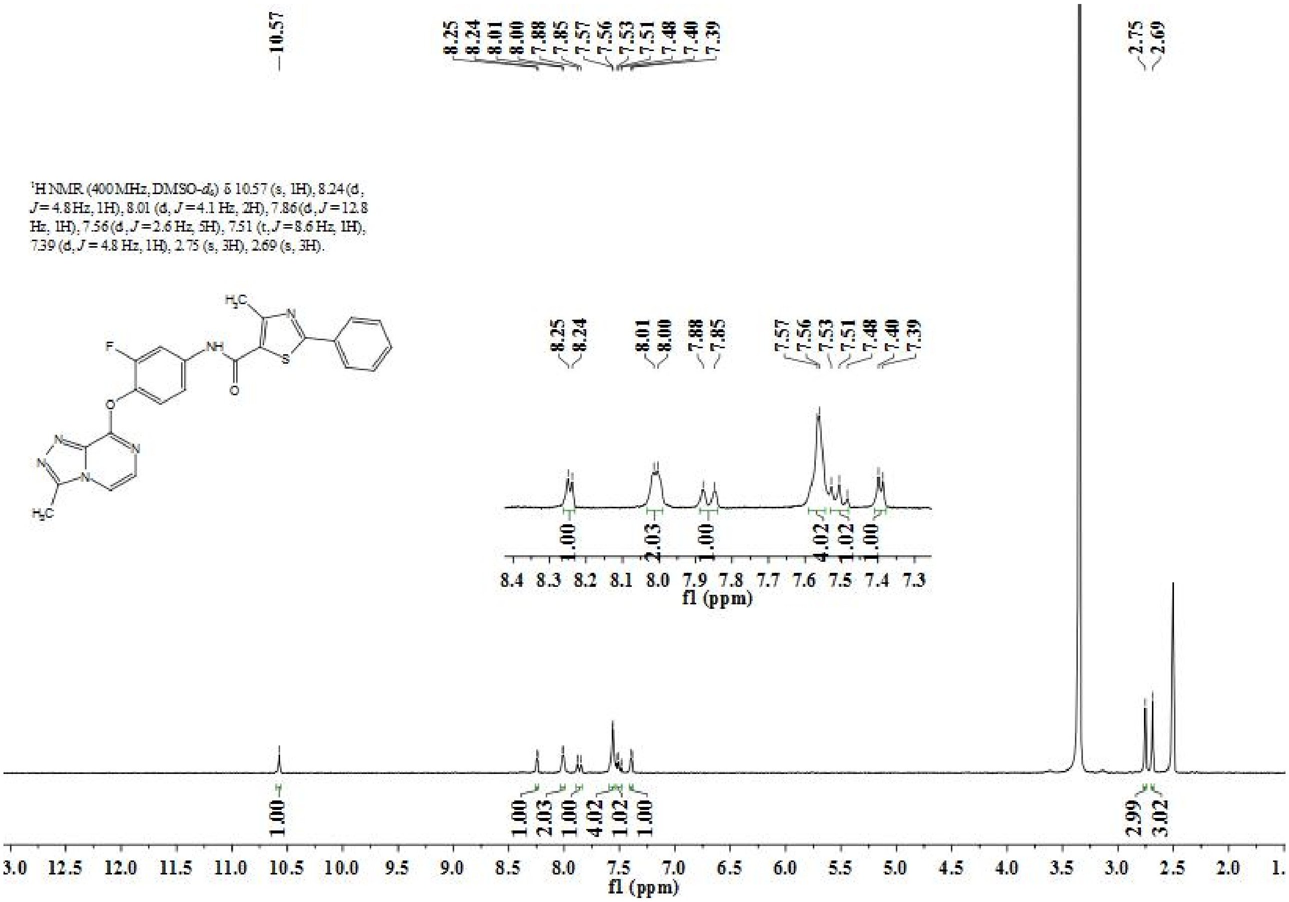

Figure 20. ^{1}H NMR spectra of compound **17h**

N-(3-fluoro-4-((3-methyl-[1,2,4]triazolo[4,3-*a*]pyrazin-8-yl)oxy)phenyl)-4-methyl-2-(pyridin-3-yl)thiazole-5-carboxamide (17i)

Light yellow solid in 49.6%, M.P.: 220.7–223.1 °C. ^{1}H NMR (400 MHz, DMSO-d_6) δ 10.43 (s, 1H), 9.21 (s, 1H), 8.63 (s, 1H), 8.29 (d, J = 5.5 Hz, 2H), 7.80 (d, J = 13.6 Hz, 2H), 7.52 (d, J = 8.2 Hz, 2H), 7.35 (s, 1H), 2.70 (s, 3H), 2.65 (s, 3H). TOF MS ES+ (m/z): $[M + H]^+$, calcd for $C_{22}H_{16}FN_7O_2S$: 462.1148, found, 462.1215.

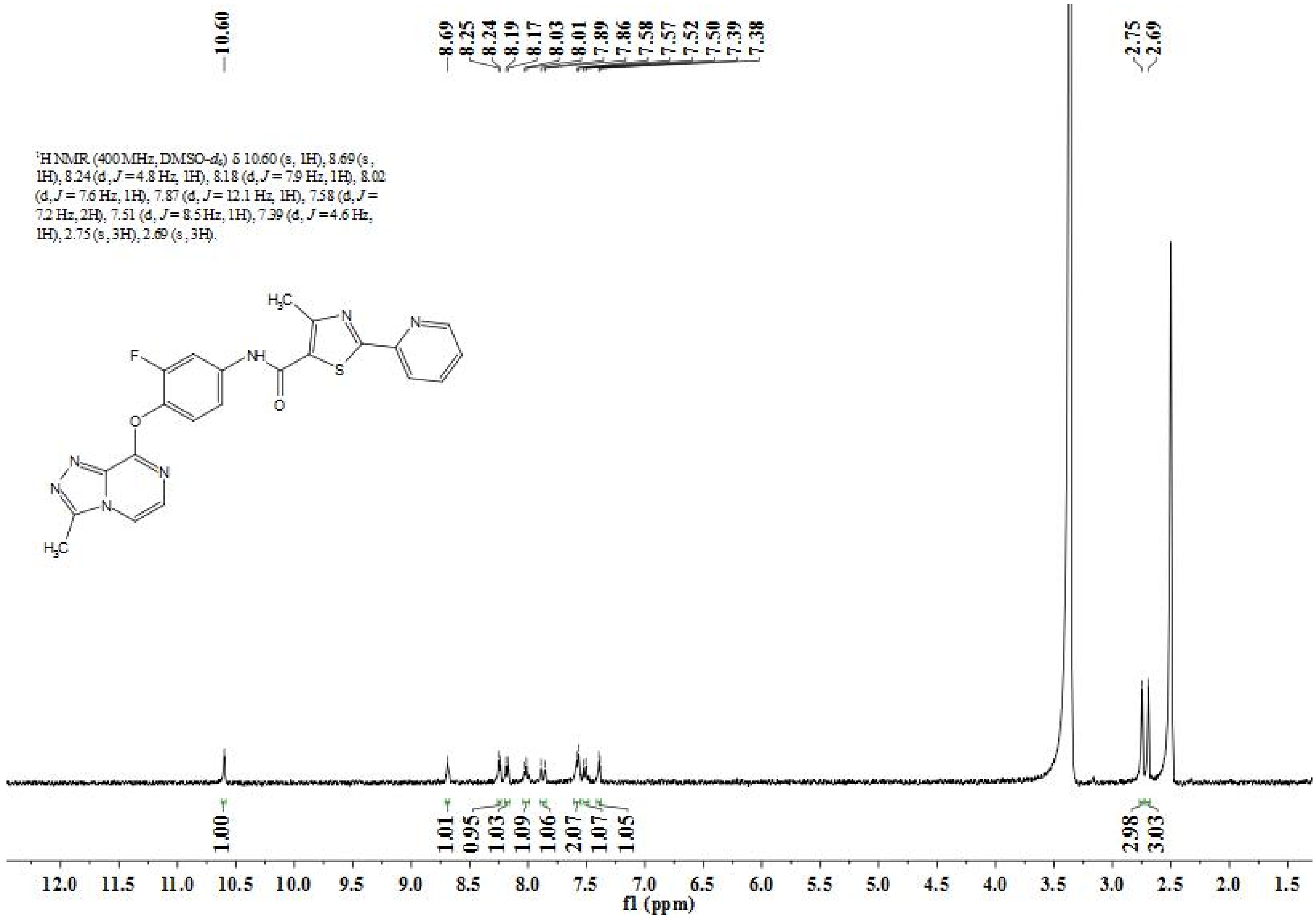

Figure 21. ^{1}H NMR spectra of compound **17i.**

N-(3-fluoro-4-((3-methyl-[1,2,4]triazolo[4,3-*a*]pyrazin-8-yl)oxy)phenyl)-4-methyl-2-(pyridin-4-yl)thiazole-5-carboxamide (17j)

Yellow solid in 41.5%, M.P.: 227.9–230.4 °C. ^{1}H NMR (400 MHz, DMSO-d_6) δ 10.68 (s, 1H), 8.74 (d, J = 5.1 Hz, 2H), 8.26 (d, J = 4.9 Hz, 2H), 7.92 (d, J = 6.1 Hz, 2H), 7.62 (d, J = 9.0 Hz, 1H), 7.41 (d, J = 4.9 Hz, 1H), 7.37 (d, J = 4.8 Hz, 1H), 2.69 (s, 3H), 2.26 (s, 3H). TOF MS ES+ (m/z): [M + H]$^+$, calcd for $C_{22}H_{16}FN_7O_2S$: 462.1148, found, 462.1070.

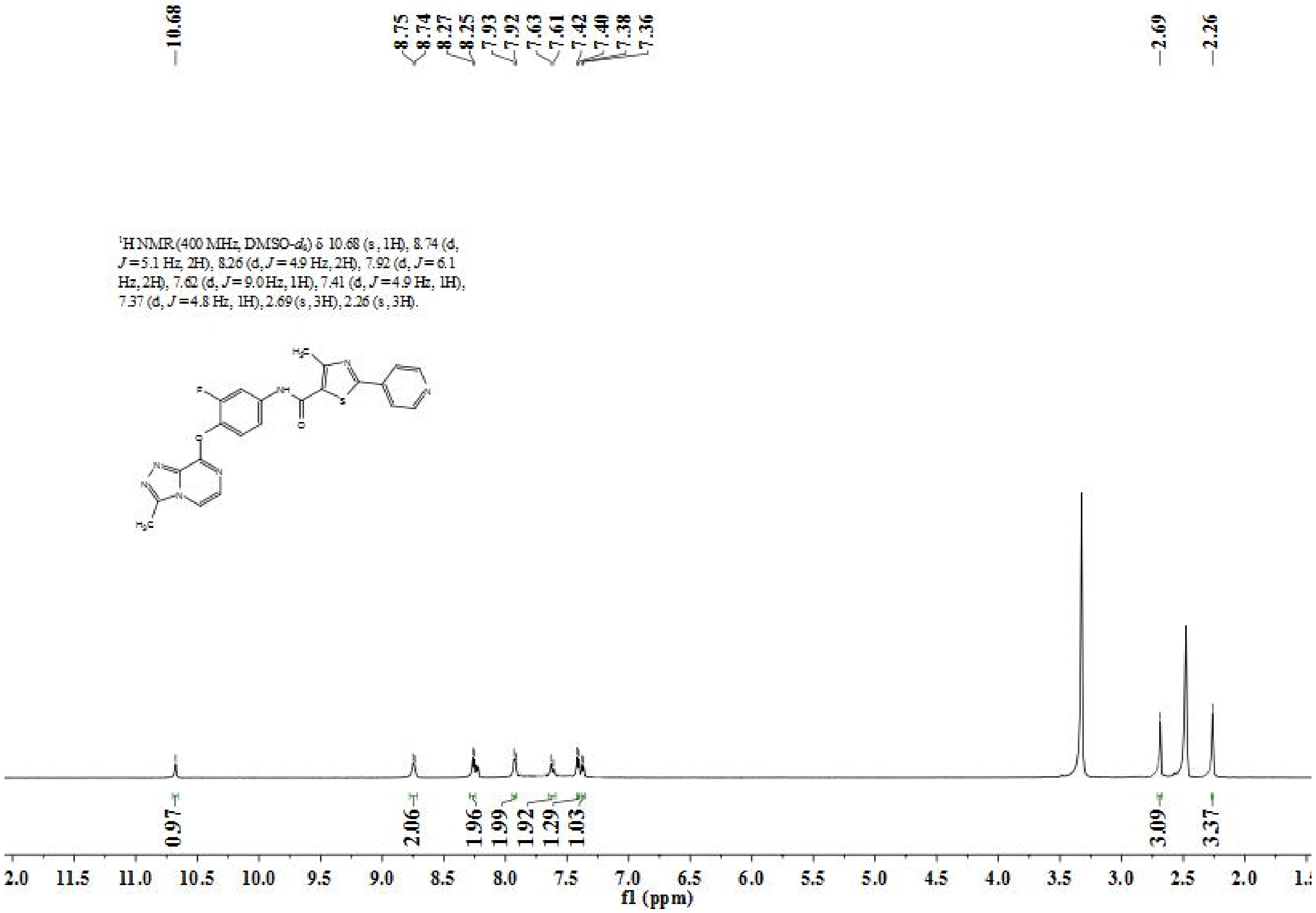

Figure 22. ^{1}H NMR spectra of compound **17j.**

N-(3-fluoro-4-((3-methyl-[1,2,4]triazolo[4,3-*a*]pyrazin-8-yl)oxy)phenyl)-2-(4-fluorophenyl)-4-methylthiazole-5-carboxamide (17k)

Yellow solid in 53.8%, M.P.: 282.3–284.7 °C. ^{1}H NMR (400 MHz, DMSO-d_6) δ 10.61 (s, 1H), 8.24 (d, J = 4.0 Hz, 1H), 8.05 (q, J = 7.6 Hz, 2H), 7.86 (d, J = 12.7 Hz, 1H), 7.54 (dd, J = 21.2, 8.5 Hz, 2H), 7.38 (p, J = 9.4, 8.9 Hz, 3H), 2.75 (s, 3H), 2.67 (s, 3H). TOF MS ES+ (m/z): [M + H]$^+$, calcd for $C_{23}H_{16}F_2N_6O_2S$: 479.1102, found, 479.1024.

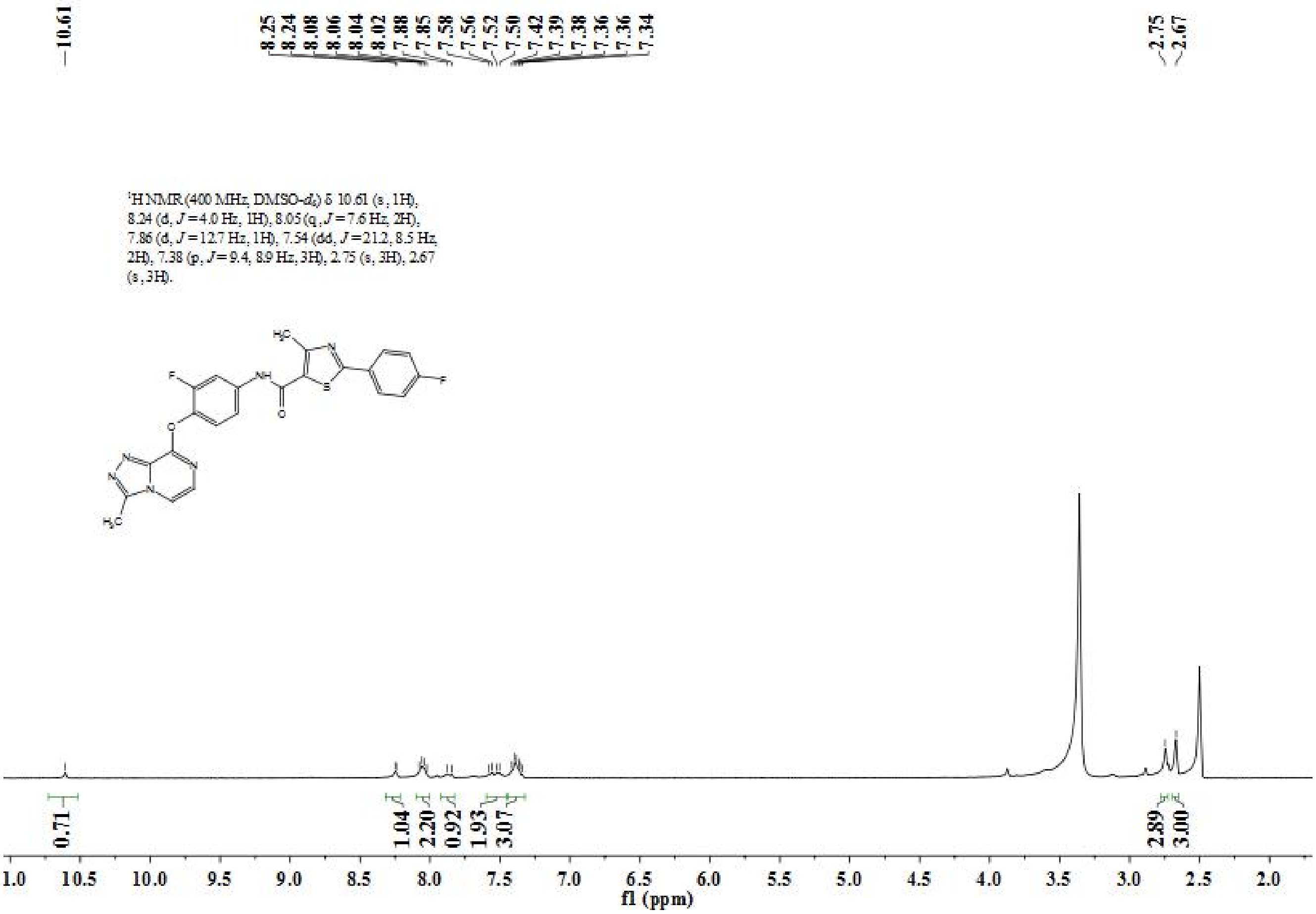

Figure 23. ^{1}H NMR spectra of compound **17k.**

1-(4-chlorophenyl)-N-(3-fluoro-4-((3-methyl-[1,2,4]triazolo[4,3-*a*]pyrazin-8-yl)oxy)phenyl)-5-(trifluoromethyl)-1H-pyrazole-4-carboxamide (17l)

Light yellow solid in 46.3%, M.P.: 238.4–240.3 °C. ^{1}H NMR (400 MHz, DMSO-d_6) δ 10.85 (s, 1H), 8.38 (s, 1H), 8.24 (d, *J* = 5.0 Hz, 1H), 7.88 (d, *J* = 12.6 Hz, 1H), 7.70 (d, *J* = 8.0 Hz, 2H), 7.62 (d, *J* = 8.4 Hz, 2H), 7.53 (d, *J* = 12.5 Hz, 2H), 7.38 (d, *J* = 4.8 Hz, 1H), 2.75 (s, 3H). TOF MS ES+ (*m/z*): [M + H]$^+$, calcd for $C_{23}H_{14}ClF_4N_7O_2$: 532.0912, found, 532.0834.

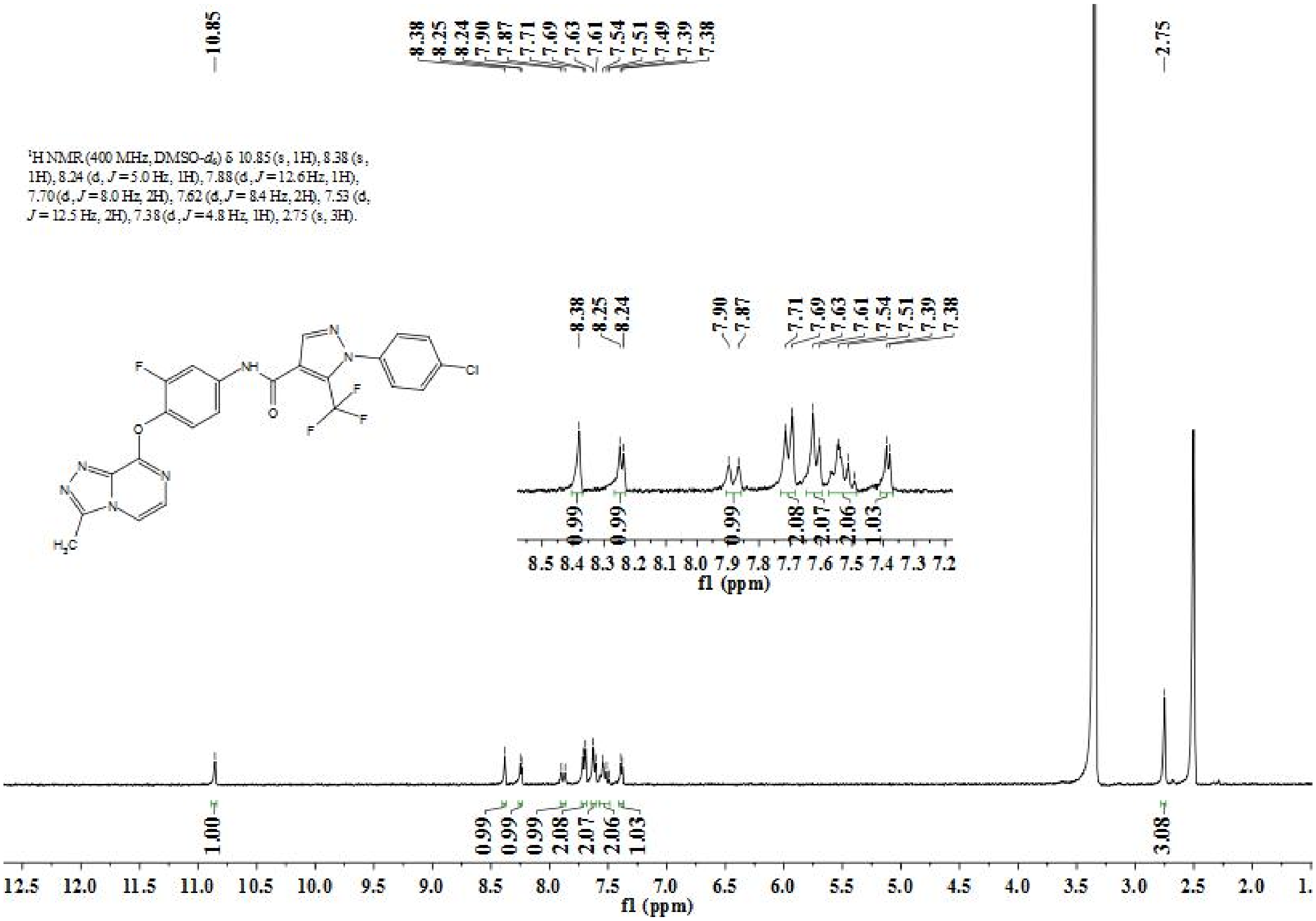

Figure 24. ^{1}H NMR spectra of compound **17l.**

N-(3-fluoro-4-((3-methyl-[1,2,4]triazolo[4,3-*a*]pyrazin-8-yl)oxy)phenyl)-3-(thiophen-2-yl)isoxazole-5-carboxamide (17m)

Yellow solid in 34.6%, M.P.: 276.9–278.1 °C. ^{1}H NMR (400 MHz, DMSO-*d*$_6$) δ 10.99 (s, 1H), 8.15 (d, *J* = 4.6 Hz, 1H), 7.88–7.81 (m, 2H), 7.78 (s, 1H), 7.64 (d, *J* = 8.9 Hz, 1H), 7.45 (t, *J* = 9.0 Hz, 1H), 7.32–7.27 (m, 2H), 7.22 (s, 1H), 2.67 (s, 3H). TOF MS ES+ (*m/z*): [M + H]$^{+}$, calcd for $C_{20}H_{13}FN_6O_3S$: 437.0832, found, 437.0754.

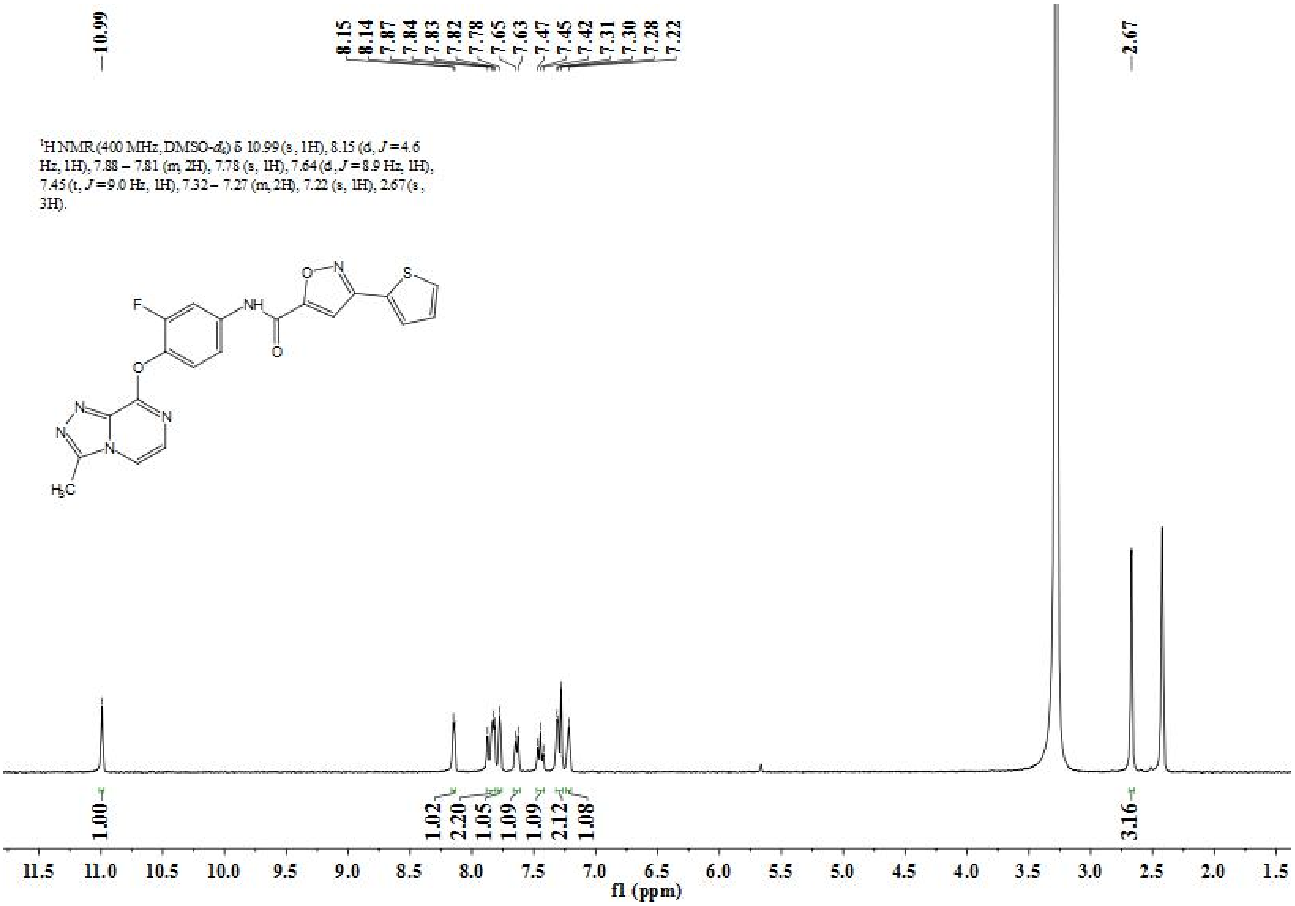

Figure 25. ^{1}H NMR spectra of compound **17m.**
